# Supplementary material for: Efficient ammonia synthesis from the air using tandem non-thermal plasma and electrocatalysis at ambient conditions
Source: Nat Commun. 2024 Apr 25;15:3524. doi: 10.1038/s41467-024-47765-9 (PMC11045753; doi:10.1038/s41467-024-47765-9)
Supplement: Supplementary file 1 — Supplementary Information [file 41467_2024_47765_MOESM1_ESM.pdf]

Supporting Information

Efficient ammonia synthesis from the air using tandem non-thermal plasma and electrocatalysis at ambient conditions

Wei Liu[1], Mengyang Xia[1], Chao Zhao[1], Ben Chong[1], Jiahe Chen[1], He Li[1], Honghui Ou[1], Guidong Yang[1]*

Affiliations:

[1]A XJTU-Oxford International Joint Laboratory for Catalysis, School of Chemical Engineering and Technology, Xi'an Jiaotong University, Xi'an, Shaanxi 710049, China.

*Corresponding author. E-mail: guidongyang@xjtu.edu.cn

Contents

Supplementary Figure 1. Open circuit potential recorded during $Ni(OH)_x$ deposition in a three-electrode cell.
Supplementary Figure 2. Pourbaix diagrams.
Supplementary Figure 3. SEM images of the electrodes at the different preparation stage.
Supplementary Figure 4. TEM and HRTEM characterization of the $Ni(OH)_x/CuO$ catalyst.
Supplementary Figure 5. Structural information of the $Ni(OH)_x/CuO$ catalyst.
Supplementary Figure 6. Structural information of the $Ni(OH)_x/Cu$ catalyst.
Supplementary Figure 7. XRD patterns of electrodes with different deposition time of the nickel species.
Supplementary Figure 8. XPS spectra of $Ni(OH)_x/CuO$ and $Ni(OH)_x/Cu$ electrodes.
Supplementary Figure 9. Raman spectra of $Ni(OH)_x/CuO$ and $Ni(OH)_x/Cu$ electrode obtained by prolonging the $Ni(OH)_x/Cu$ deposition time to 5 h.
Supplementary Figure 10. Selected area electron diffraction (SAED) of $Ni(OH)_x/Cu$.
Supplementary Figure 11. EXAFS spectra in k-space.
Supplementary Figure 12. Fitting results of the EXAFS spectra at k-space and R space.
Supplementary Figure 13. Characterization of as-synthesized $Ni(OH)_2$.
Supplementary Figure 14. SEM images of the $Ni(OH)_x/Cu$ electrodes with different deposition time.
Supplementary Figure 15. Calibration curve of NH_3 obtained by indophenol blue method using NH_4Cl solution as standards.
Supplementary Figure 16. Calibration curve of NO_2^- using KNO_2 solution as standards.
Supplementary Figure 17. Calibration curve of NO_3^- obtained by ion chromatography using KNO_3 solution as standards.
Supplementary Figure 18. Reference electrode calibration with respect to reversible hydrogen electrode.
Supplementary Figure 19. LSV curves and NH_3 partial current density of $Ni(OH)_2$.
Supplementary Figure 20. Faradaic efficiencies of NH_3 and NO_2^- during nitrate reduction.
Supplementary Figure 21. Nitrate reduction performance of $Ni(OH)_2$.
Supplementary Figure 22. Comparison of the ECSAs of different samples.
Supplementary Figure 23. Nitrate reduction performance of $Ni(OH)_x/Cu$ electrodes with different deposition time.
Supplementary Figure 24. Voltammograms of OH_{ad} peaks collected in an Ar-purged 1 M KOH electrolyte with a scan rate of 50 mV s^{-1}.
Supplementary Figure 25. Nitrate reduction performance of $Ni(OH)_x/CuO$ electrode.
Supplementary Figure 26. Complete nitrate conversion using $Ni(OH)_x/Cu$ with an initial 1 M KOH with 0.1 M NO_3^- (equals to 1400 mg L^{-1} NO_3^-−N) at −0.25 V vs. RHE.
Supplementary Figure 27. Evaluation of the performance of $Ni(OH)_x/Cu$ in electrolytes with different NO_3^- concentrations.
Supplementary Figure 28. NH_3 yield rate and corresponding NH_3 FE over the 15 independent tests using $Ni(OH)_x/Cu$ electrode at −0.25 V (vs. RHE) in 1 M KOH with 0.1 M NO_3^-.
Supplementary Figure 29. 10 cyclic test of the $Ni(OH)_x/Cu$ electrode.
Supplementary Figure 30. Long-term stability test in H-type flow cell.
Supplementary Figure 31. Structure characterization of the tested $Ni(OH)_x/Cu$ sample.
Supplementary Figure 32. eNO_2^-RR performance evaluation in 1 M KOH with 0.1 M KNO_2.
Supplementary Figure 33. ECSA-normalized NH_3 yield rate of Cu and $Ni(OH)_2$ in eNO_2^-RR.
Supplementary Figure 34. eNO_3^-RR performance at more negative potentials.
Supplementary Figure 35. Tafel slopes of HER and DER over Cu and $Ni(OH)_x/Cu$.

Supplementary Figure 36. Cathode shift values under different current densities over Cu and $Ni(OH)_x$/Cu electrodes in the presence/absence of NO_3^-.
Supplementary Figure 37. NH_3 partial current density with the addition of DMPO or D_2O over Cu and $Ni(OH)_x$/Cu at −0.25 V (vs. RHE).
Supplementary Figure 38. CV curves in Ar-saturated 1 M KOH with a scan rate of 100 mV s^{-1}.
Supplementary Figure 39. DMPO-involved EPR spectra of the solutions obtained after 10 min of eNO_3^-RR test over Cu and $Ni(OH)_x$/Cu.
Supplementary Figure 40. Atomic configurations of water adsorption and dissociation on Cu in the presence of different cation hydrates.
Supplementary Figure 41. Atomic configurations of water adsorption and dissociation on $Ni(OH)_x$/Cu in the presence of different cation hydrates.
Supplementary Figure 42. Cation−water coordination number determination.
Supplementary Figure 43. Snapshots of MD simulation.
Supplementary Figure 44. HER performance evaluation in the presence of different alkali metal cations.
Supplementary Figure 45. *In situ* electrochemical ATR-SEIRAS.
Supplementary Figure 46. Geometries of the molecular structures for DFT calculations.
Supplementary Figure 47. Photograph of the experimental set-up for *in situ* Raman measurement.
Supplementary Figure 47. Parameters of the spark discharge NTP.
Supplementary Figure 48. The spark discharge NTP configuration.
Supplementary Figure 49. Mass spectra of the feeding gas with or without spark discharge NTP.
Supplementary Figure 50. Effect of different spark discharge NTP conditions on NO_x^- concentration in 25 mL of 1 M KOH absorbing solution for 10 min of operation.
Supplementary Figure 51. Stability evaluation of the spark discharge NTP.
Supplementary Figure 52. Comparison of NH_3 yield under different operation models.
Supplementary Figure 53. Configuration of the pNOR-eNO_x^-RR system for batch experiments.
Supplementary Figure 54. Configuration of the pNOR-eNO_x^-RR system for long-term continuous operation.
Supplementary Figure 55. Concentration variations of NH_3 and NO_x^-.
Supplementary Figure 56. Electrochemical impedance spectroscopy (EIS) of $Ni(OH)_x$/Cu obtained at different operation time.
Supplementary Figure 57. pH variations of the catholyte in pNOR-eNO_x^-RR tandem system and absorption solution of pNOR without eNO_x^-RR.
Supplementary Figure 58. EIS obtained in the absorption solution of pNOR without eNO_x^-RR.
Supplementary Figure 59. Long-term continuous test of pNOR-eNO_x^-RR system using Cu electrode.
Supplementary Figure 60. NH_3 products collection.
Supplementary Figure 61. Ammonia products characterization.
Supplementary Figure 62. Thermodynamic cycle employed to calculate the overall free energy of NO_3^- adsorption.
Supplementary Table 1. EXAFS fitting parameters at the Ni *K*-edge for various samples.
Supplementary Table 2. ICP-MS data for the $Ni(OH)_x$/Cu nanowires that ultrasonic peeling from the Cu skeleton and $Ni(OH)_x$/Cu bulk electrodes with different deposition times.
Supplementary Table 3. Comparison of NH_3 yield rate and Faradaic efficiency of this work with previously reported works covering pathways of pNOR-eNO_x^-RR, eNRR, and Li-mediated eNRR.
Supplementary Table 4. Enthalpy (ΔH_f^o) and Gibbs free energy (ΔG_f^o) of the reactants and products.
Supplementary Note 1. Energy efficiency calculation of pNOR-eNO_x^-RR.
Supplementary Note 2. Detials for the DFT calculations.

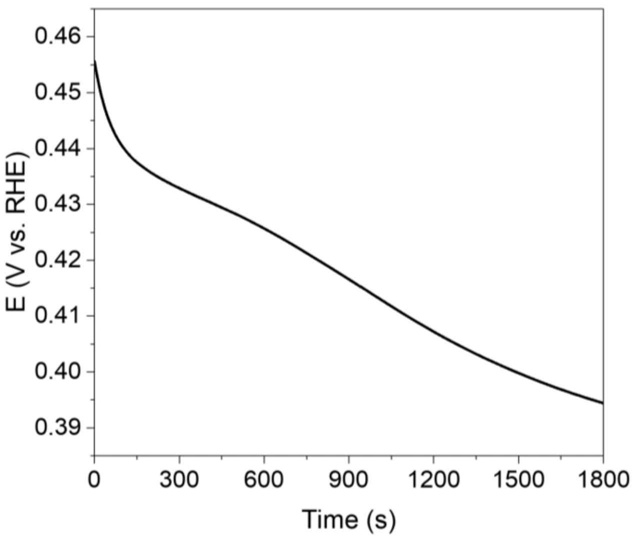

Supplementary Figure 1 | Open circuit potential (OCP) recorded during $Ni(OH)_x$ deposition in a three-electrode cell. The OCP profile was measured in a single cell with Ag/AgCl electrode and Pt plate as the reference electrode and counter electrode, respectively. The OCP varied during the $Ni(OH)_x$ deposition process.

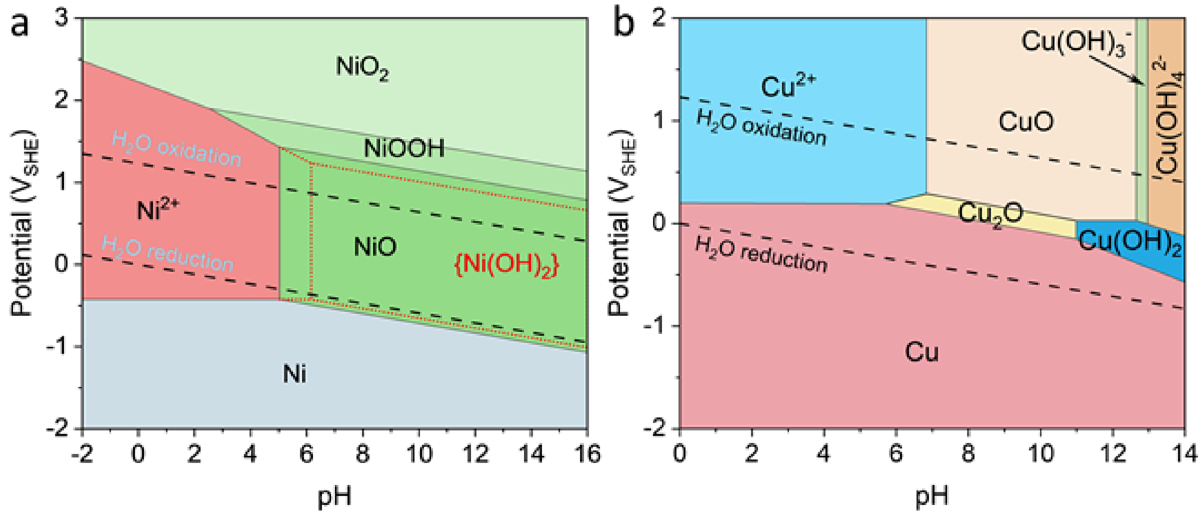

Supplementary Figure 2 | Pourbaix diagrams. **a.** Ni. **b.** Cu. CuO is easily reduced to the metallic state of Cu, while the reduction of nickel hydroxide to Ni exhibits more negative potential at the same pH. Thus, by meticulously controlling the CV scan range from 0.2 to −0.3 V vs. RHE, the CuO substrate in $Ni(OH)_x$/CuO can be fully reduced to Cu during the prereduction process with $Ni(OH)_x$ remaining unchanged.

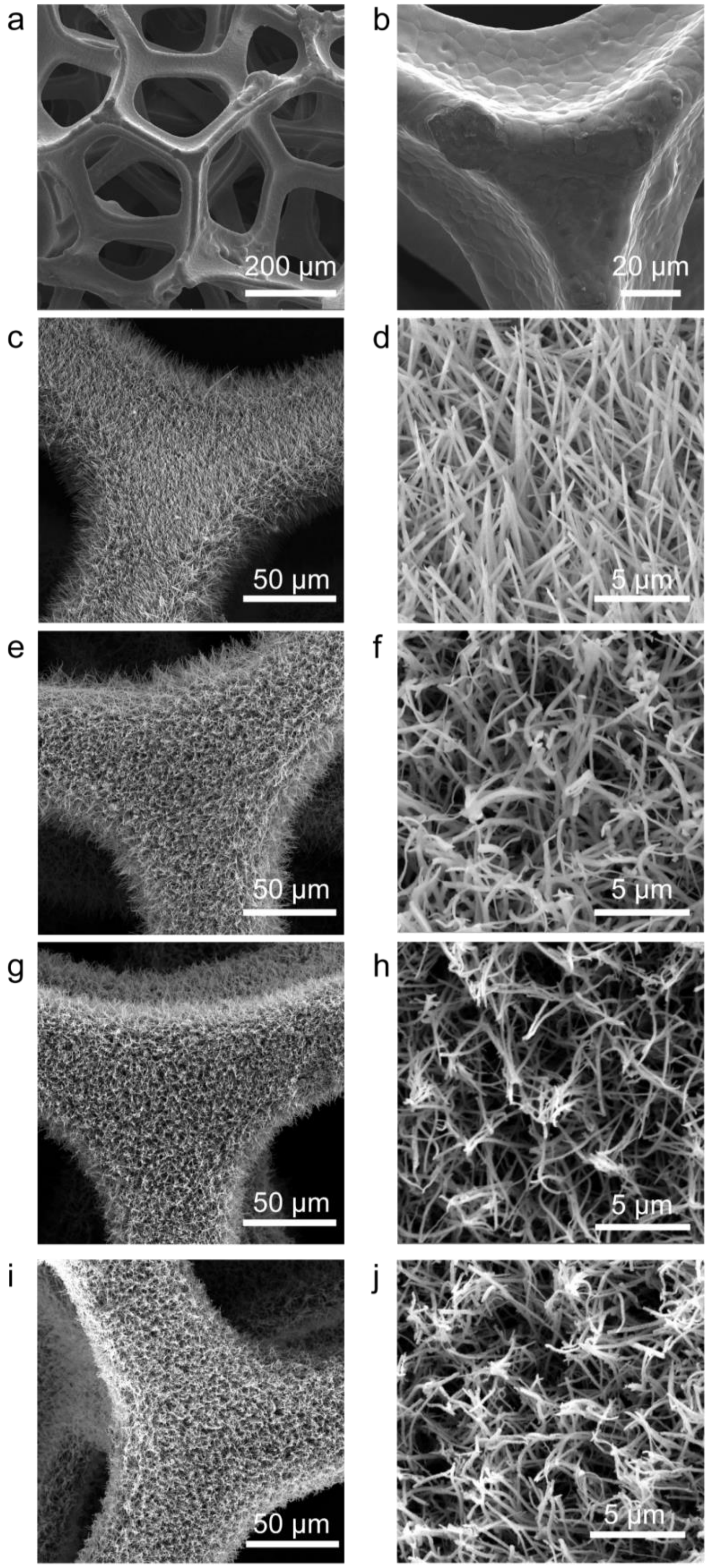

Supplementary Figure 3 | SEM images of the electrodes at the different preparation stage. a-b. Cu foam. **c-d.** $Cu(OH)_2$ NWA. **e-f.** CuO NWA. **g-h.** $Ni(OH)_x$/CuO NWA. **i-j.** $Ni(OH)_x$/Cu NWA.

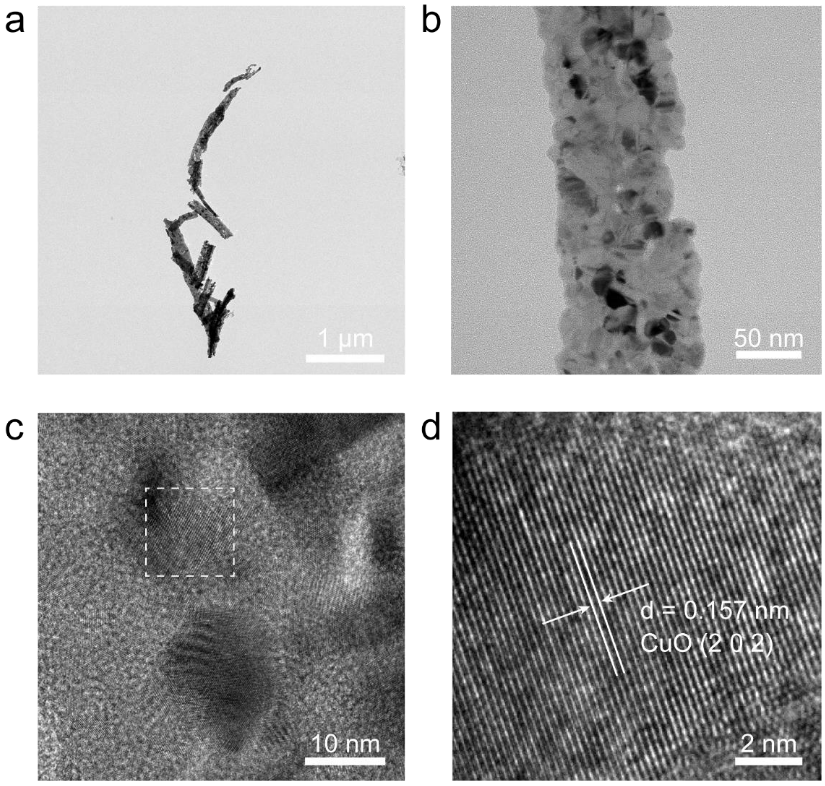

Supplementary Figure 4 | TEM and HRTEM characterization of the Ni(OH)$_x$/CuO catalyst. a-b. TEM images of the Ni(OH)$_x$/CuO nanowire. **c.** HRTEM image. **d.** Indication of CuO (202) facet with the lattice spacing of 1.57 Å.

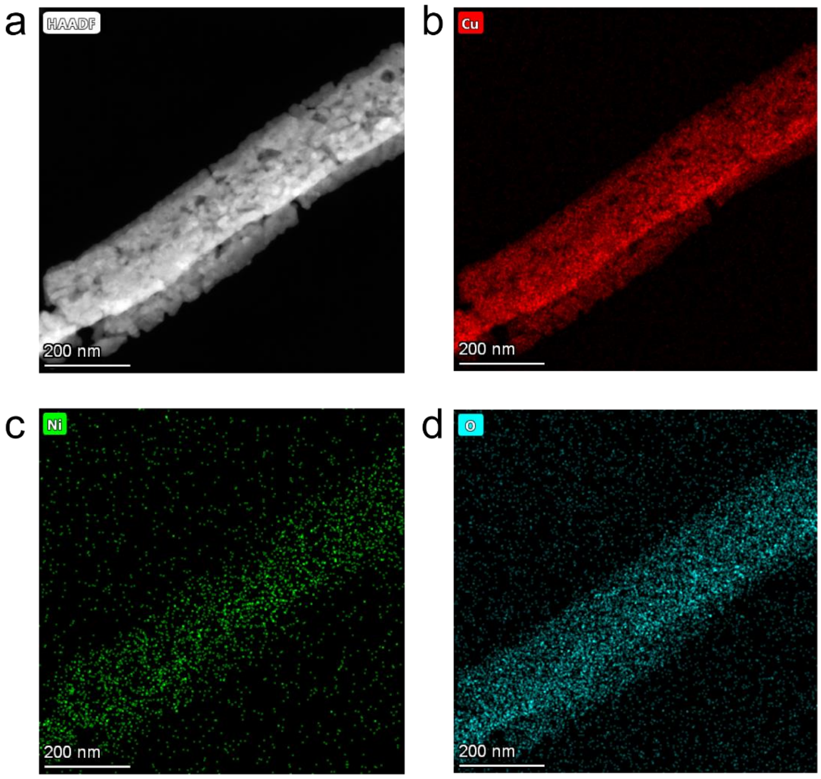

Supplementary Figure 5 | Structural information of the Ni(OH)$_x$/CuO catalyst. a. HAADF-STEM image. **b-d.** The corresponding EDS images of Cu, Ni, and O elements.

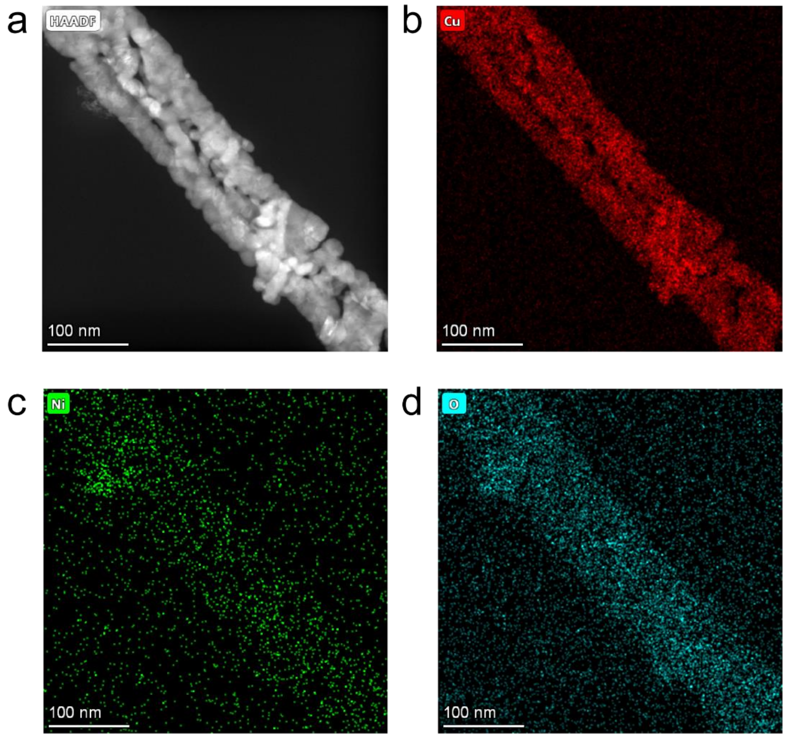

Supplementary Figure 6 | Structural information of the $Ni(OH)_x/Cu$ catalyst. a. HAADF-STEM image. **b-d.** The corresponding EDS images of Cu, Ni, and O elements.

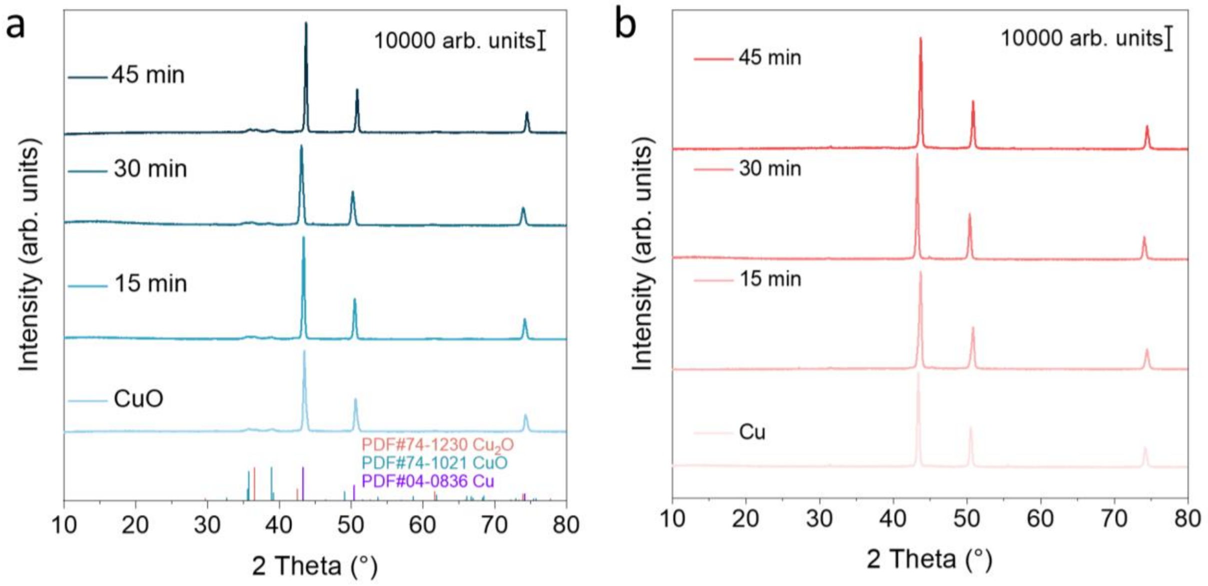

Supplementary Figure 7 | XRD patterns of electrodes with different deposition time of the nickel species. a. Electrodes before the CV prereduction. **b.** Electrodes after the CV prereduction.

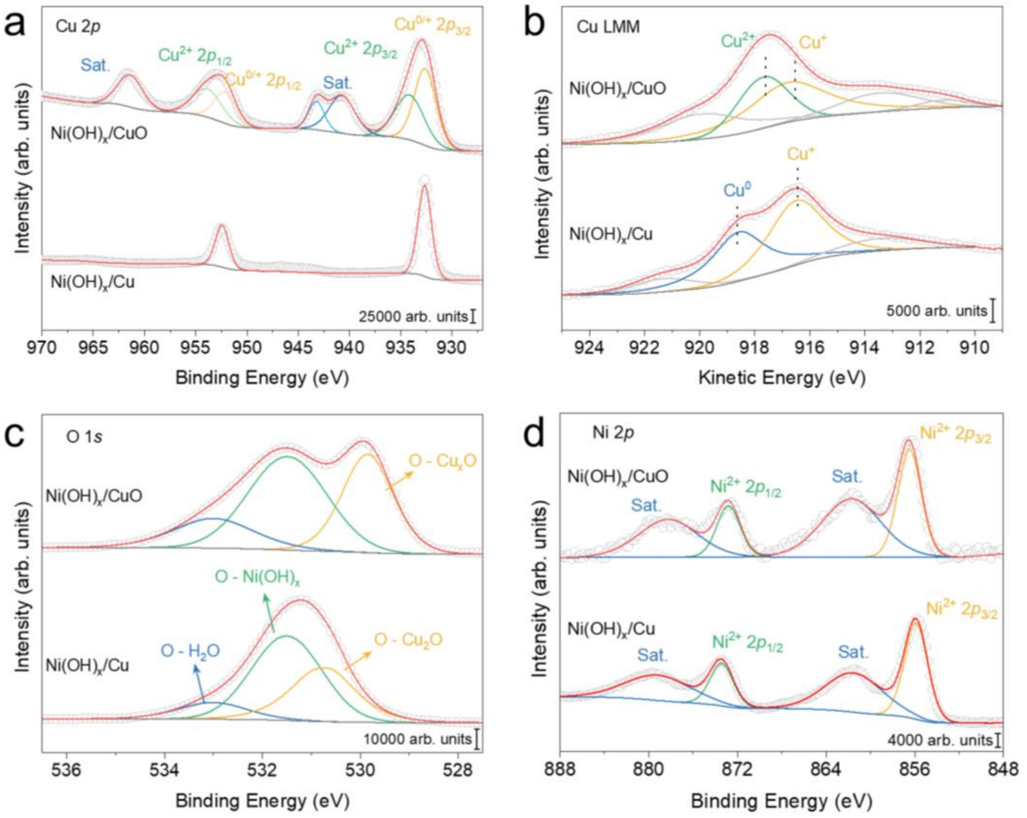

Supplementary Figure 8 | XPS spectra of $Ni(OH)_x/CuO$ and $Ni(OH)_x/Cu$ electrodes. a. High-resolution Cu 2*p* spectra. **b.** Cu LMM Auger spectra. **c.** O 1*s* spectra. **d.** High-resolution Ni 2*p* spectra.

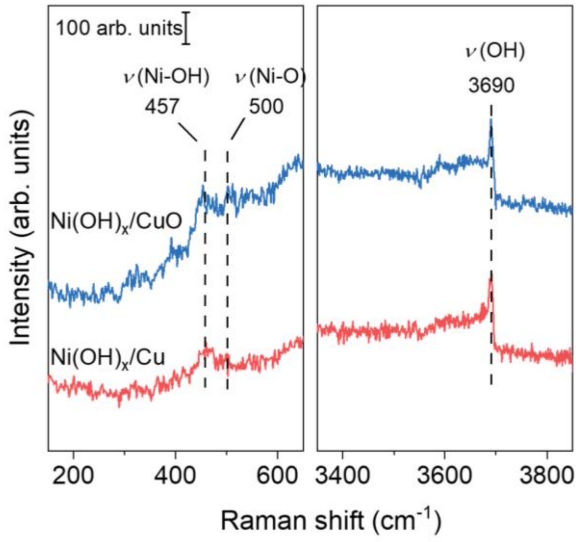

Supplementary Figure 9 | Raman spectra of $Ni(OH)_x/CuO$ and $Ni(OH)_x/Cu$ electrode obtained by prolonging the $Ni(OH)_x/Cu$ deposition time to 5 h.

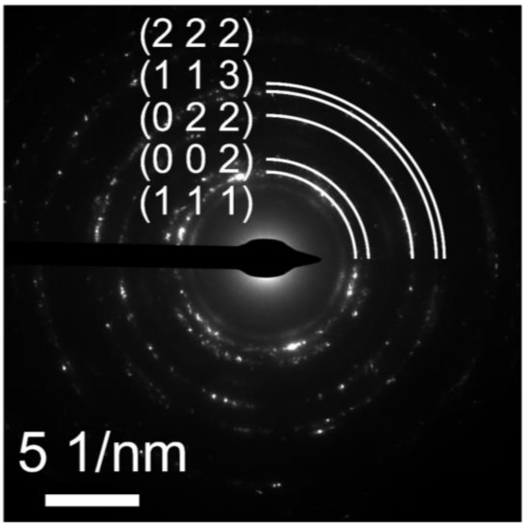

Supplementary Figure 10 | Selected area electron diffraction (SAED) of $Ni(OH)_x/Cu$. The concentric ring-like structure reveals that the sample is polycrystalline. However, these diffraction circles are all assigned to the (2 2 2), (1 1 3), (0 2 2), (0 0 2), and (1 1 1) facets of the metallic Cu. No diffraction circle of $Ni(OH)_2$ is observed, indicating that the nickel species on Cu may exist with an amorphous structure.

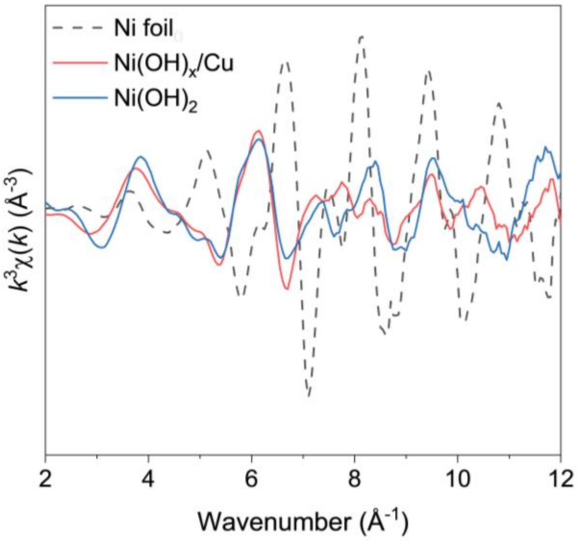

Supplementary Figure 11 | EXAFS spectra in k-space. EXAFS spectra in k-space provide valuable information about the local atomic structure surrounding the absorbing atom. The oscillations arise from the interference between the outgoing photoelectron wave and the waves scattered by neighboring atoms. $Ni(OH)_x/Cu$ and $Ni(OH)_2$ standard samples exhibit similar oscillations, indicating a similar coordination environment around the Ni atom.

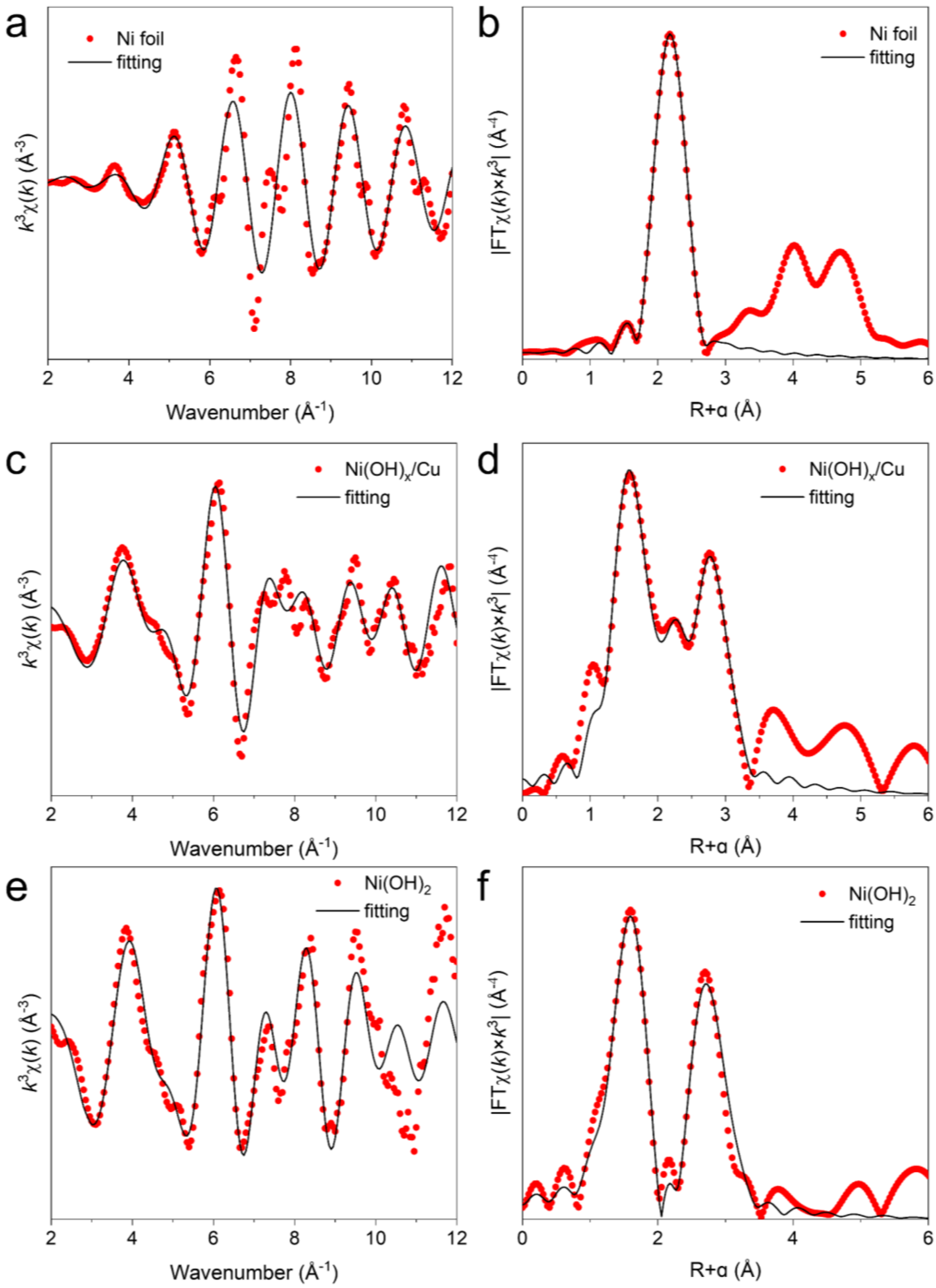

Supplementary Figure 12 | Fitting results of the EXAFS spectra at k-space and R space. a-b. Ni foil. c-d. $Ni(OH)_x/Cu$. e-f. $Ni(OH)_2$.

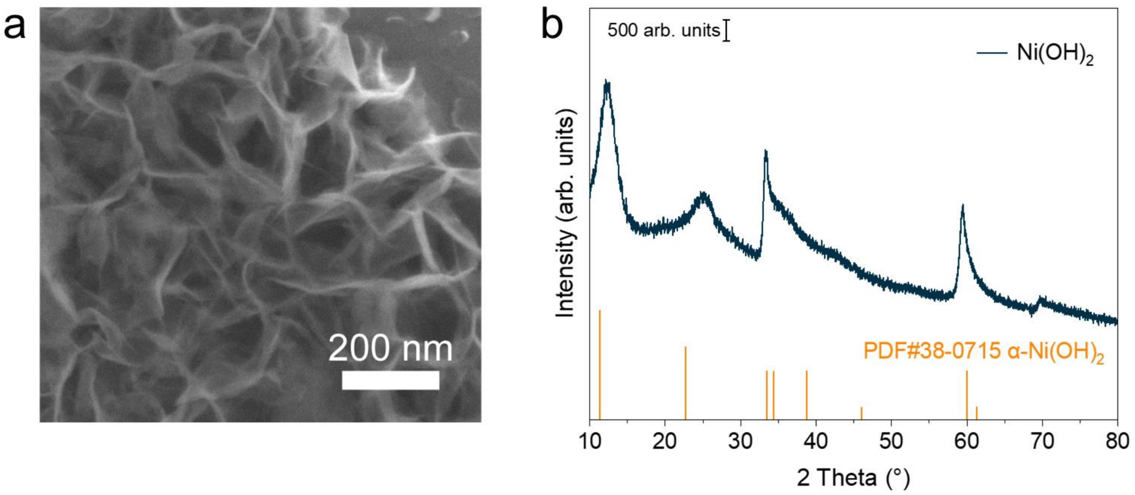

Supplementary Figure 13 | Characterization of as-synthesized $Ni(OH)_2$. a. SEM image. **b.** XRD pattern. The as-synthesized $Ni(OH)_2$ exhibits nanosheet morphology. According to the XRD pattern, the as-synthesized $Ni(OH)_2$ is α-$Ni(OH)_2$.

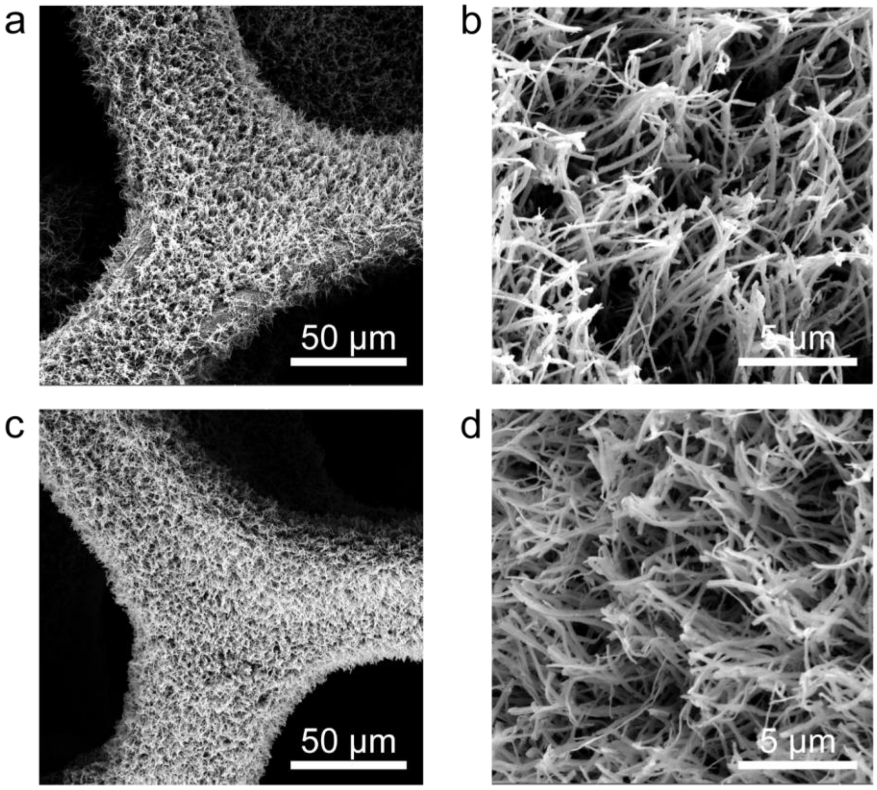

Supplementary Figure 14 | SEM images of the $Ni(OH)_x$/Cu electrodes with different deposition time. a-b. 15 min. **c-d.** 45 min.

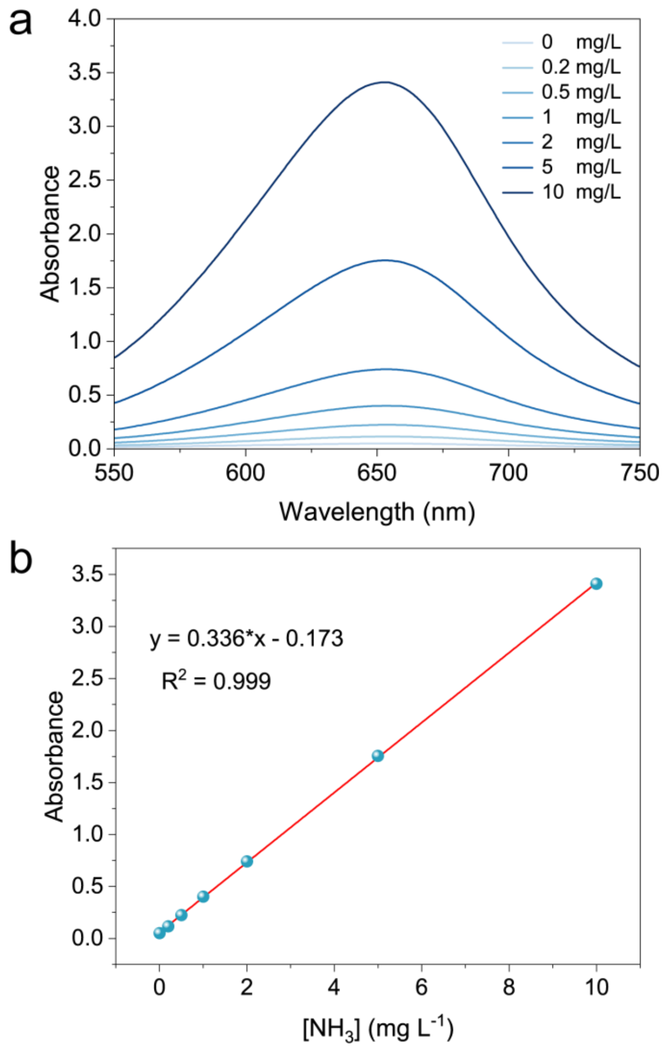

Supplementary Figure 15 | Calibration curve of NH_3 obtained by indophenol blue method using NH_4Cl solution as standards. a. UV-vis spectra of indophenol assays with various NH_4^+ concentrations. **b.** Linear fitting results of the calibration curve.

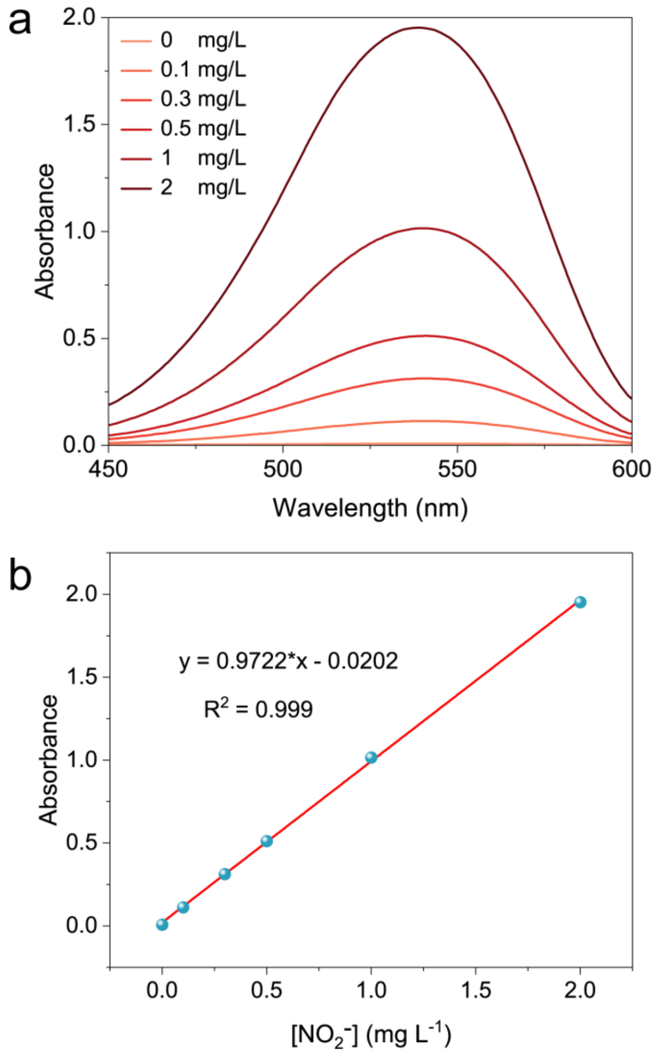

Supplementary Figure 16 | Calibration curve of NO_2^- using KNO_2 solution as standards. a. UV-vis spectra of assays with various NO_2^- concentrations. **b.** Linear fitting results of the calibration curve.

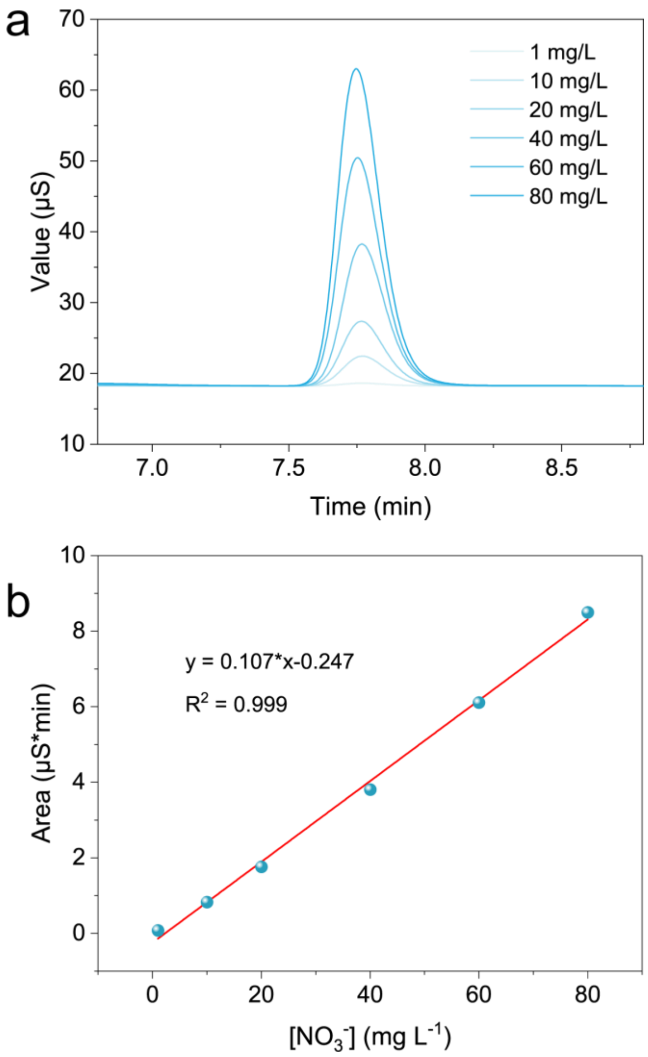

Supplementary Figure 17 | Calibration curve of NO_3^- obtained by ion chromatography using KNO_3 solution as standards. a. Electric conductance-concentration spectra of assays with various NO_3^- concentrations. **b.** Linear fitting results of plots of integral area against concentration.

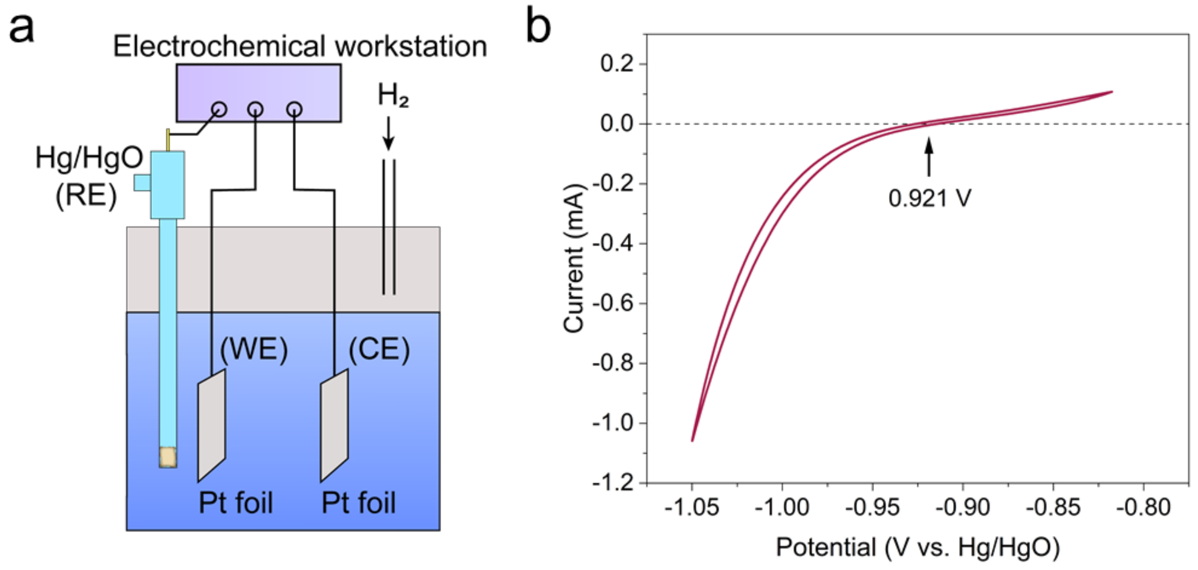

Supplementary Figure 18 | Reference electrode calibration with respect to reversible hydrogen electrode. a. Schematic of the setup for calibration of the reference electrode that used in this study. **b.** CV curve in the hydrogen saturated 1 M KOH electrolyte.

So in 1 M KOH, E (RHE) = E (Hg/HgO) + 0.921 V.

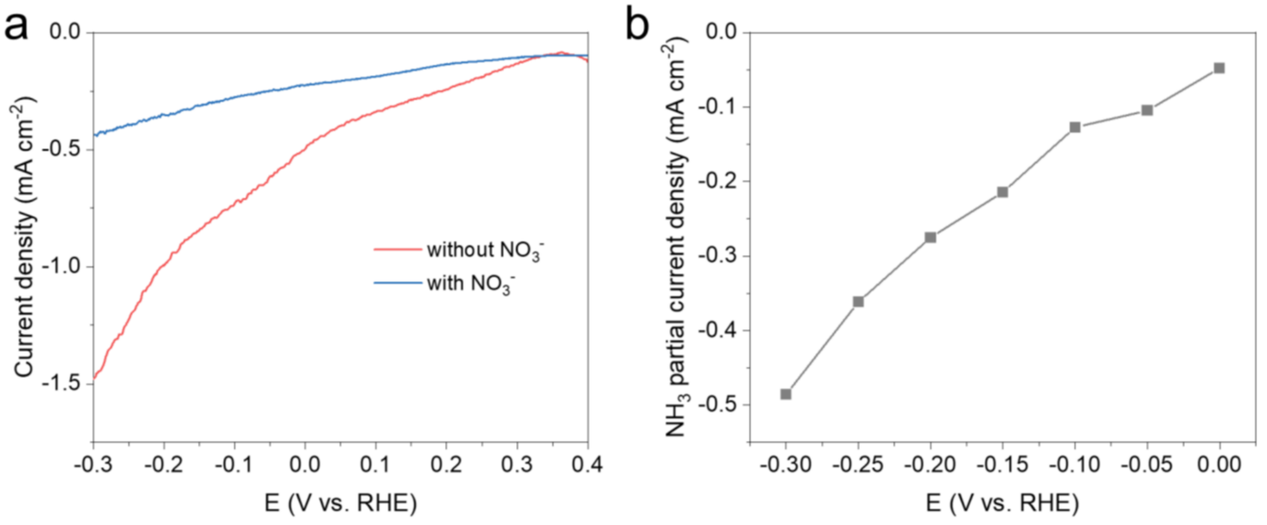

Supplementary Figure 19 | a. LSV curves of $Ni(OH)_2$ with/without NO_3^- in 1 M KOH. **b.** NH_3 partial current density over $Ni(OH)_2$ in eNO_3^-RR.

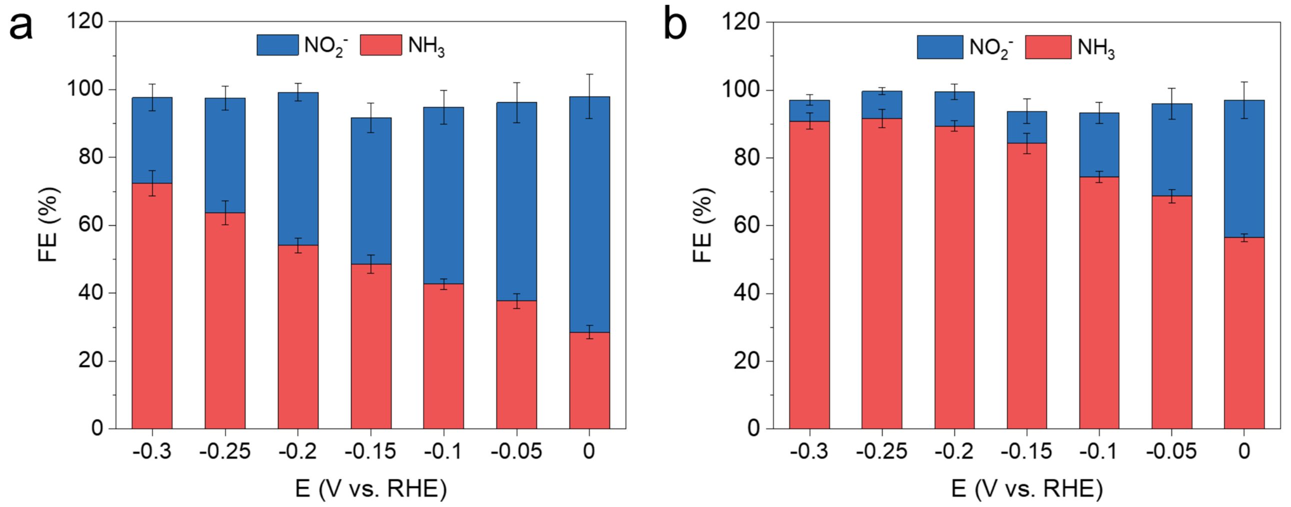

Supplementary Figure 20 | Faradaic efficiencies of NH_3 and NO_2^- during nitrate reduction. a. Using Cu as the cathode. **b.** Using $Ni(OH)_x$/Cu as the cathode. The error bars represent the standard deviation from at least three independent measurements.

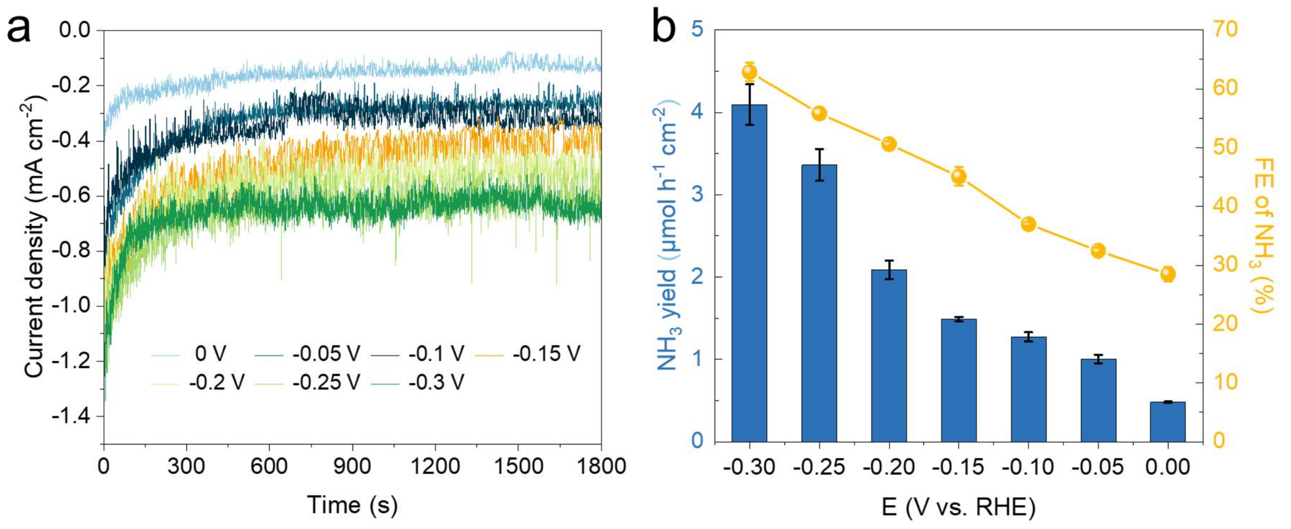

Supplementary Figure 21 | Nitrate reduction performance of $Ni(OH)_2$. a. I-t curves of eNO_3^-RR over $Ni(OH)_2$ under different applied potentials. **b.** NH_3 yield rate and corresponding FE of NH_3. The error bars represent the standard deviation from at least three independent measurements.

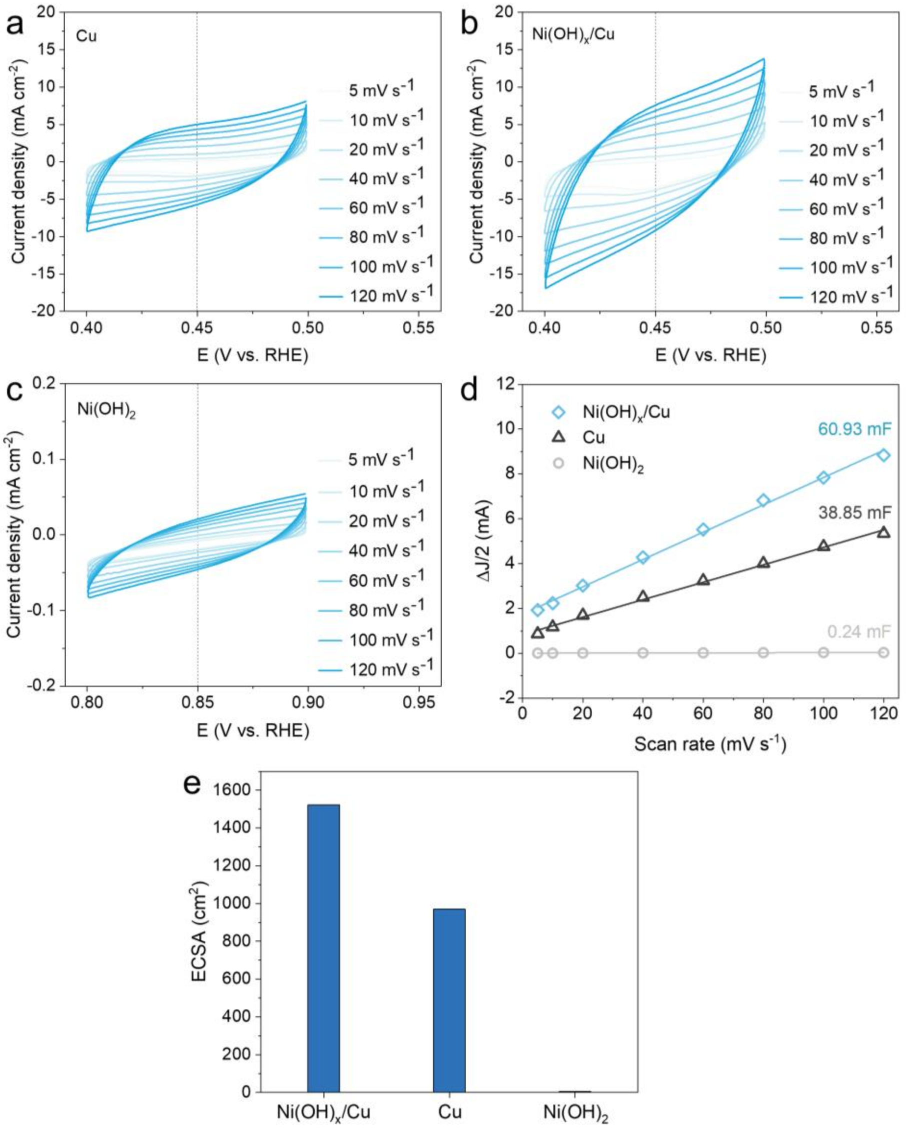

Supplementary Figure 22 | Comparison of the ECSAs of different samples. a-c. CV curves for the determination of the double−layer capacitance in Ar−saturated 1 M KOH of Cu (**a**), $Ni(OH)_x/Cu$ (**b**), and $Ni(OH)_2$ (**c**). **d.** Linear fitting of the plots of half the difference between anodic and cathodic currents against scan rate. **e.** ECSA values calculated based on a specific capacitance of 40 μF cm^{-2}.

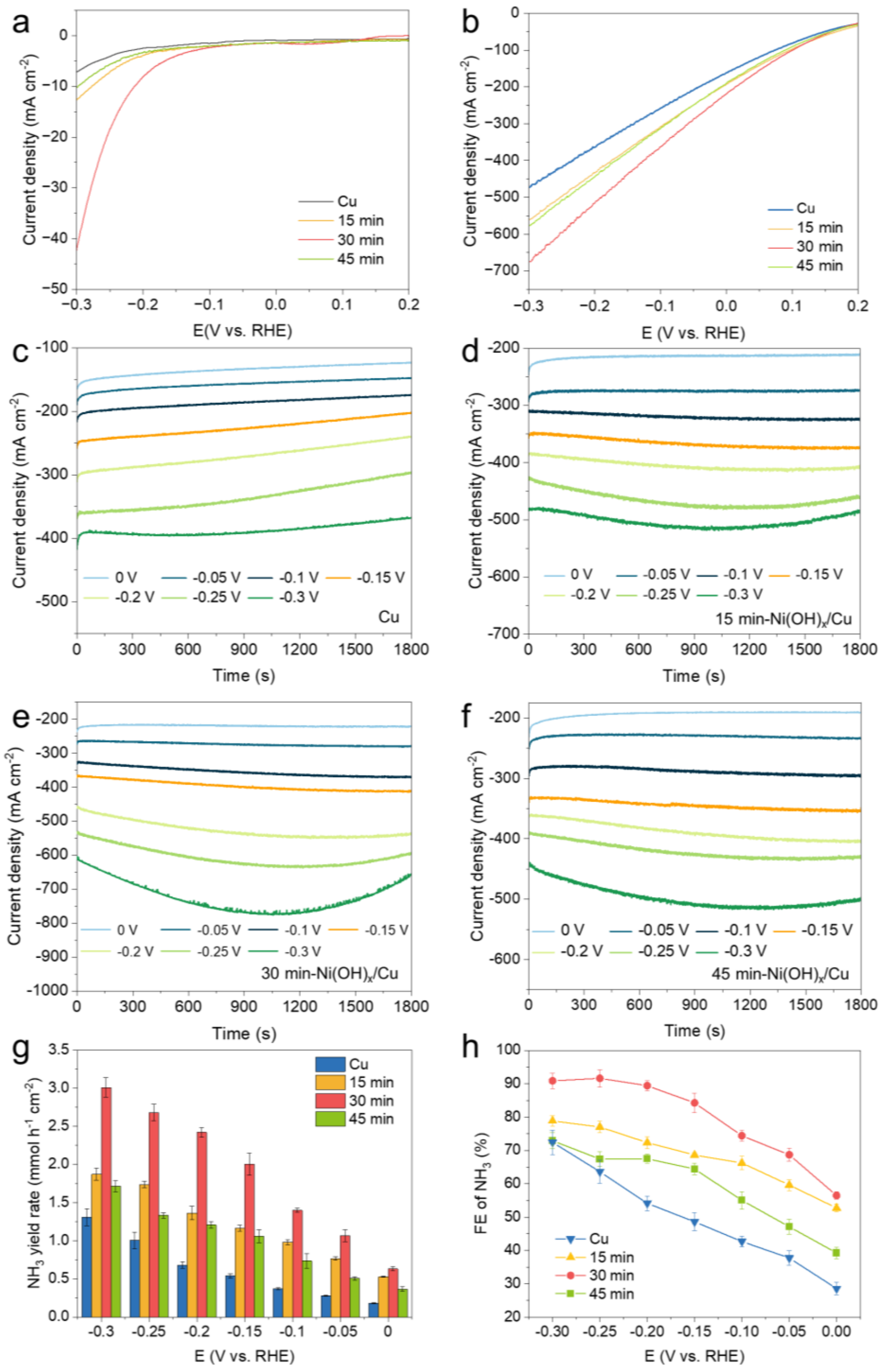

Supplementary Figure 23 | Nitrate reduction performance of $Ni(OH)_x$/Cu electrodes with different deposition time. a. LSV curves in 1 M KOH without adding NO_3^-. **b.** LSV curves in 1 M KOH with the addition of 0.1 M NO_3^-. **c-f.** I-t curves under different potentials over Cu (c) and $Ni(OH)_x$/Cu electrodes with different deposition time of 15 min (d), 30 min (e), and 45 min (f). **g.** NH_3 yield rate under different potentials. **h.** Corresponding Faradaic efficiency of NH_3. The error bars represent the standard deviation from at least three independent measurements.

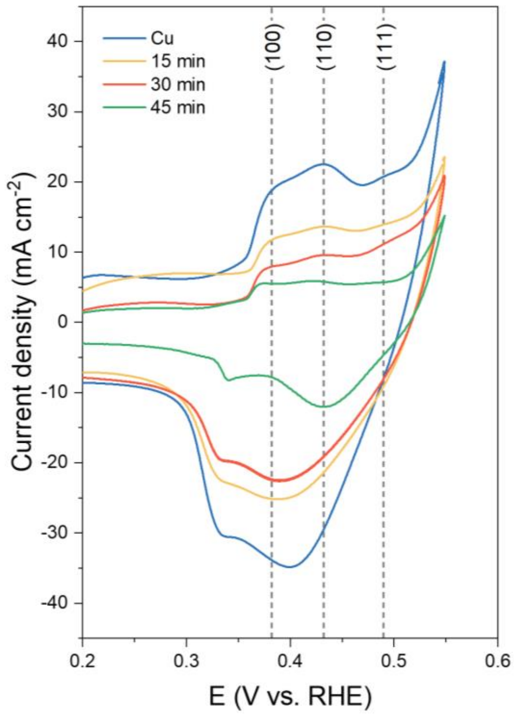

Supplementary Figure 24 | Voltammograms of OH_{ad} peaks collected in an Ar-purged 1 M KOH electrolyte with a scan rate of 50 mV s^{-1}.

Various facets of Cu single crystals exhibit distinct hydroxide electrosorption (OH_{ad}) peaks at different potentials in voltammograms, allowing for the use of OH_{ad} to probe the surface structures of polycrystalline Cu electrodes. The voltammograms measured for the Cu nanowires exhibit a series of reversible peaks in the potential region of 0.35−0.50 V, which can be assigned to the features of low-index facets of face-centered cubic (fcc) Cu (i.e., ~0.38 V for (100), ~0.43 V for (110), and ~0.49 V for (111)). As the deposition time increases, the OH_{ad} peaks gradually diminish, indicating that an increasing proportion of the Cu surface is being covered by $Ni(OH)_x$.

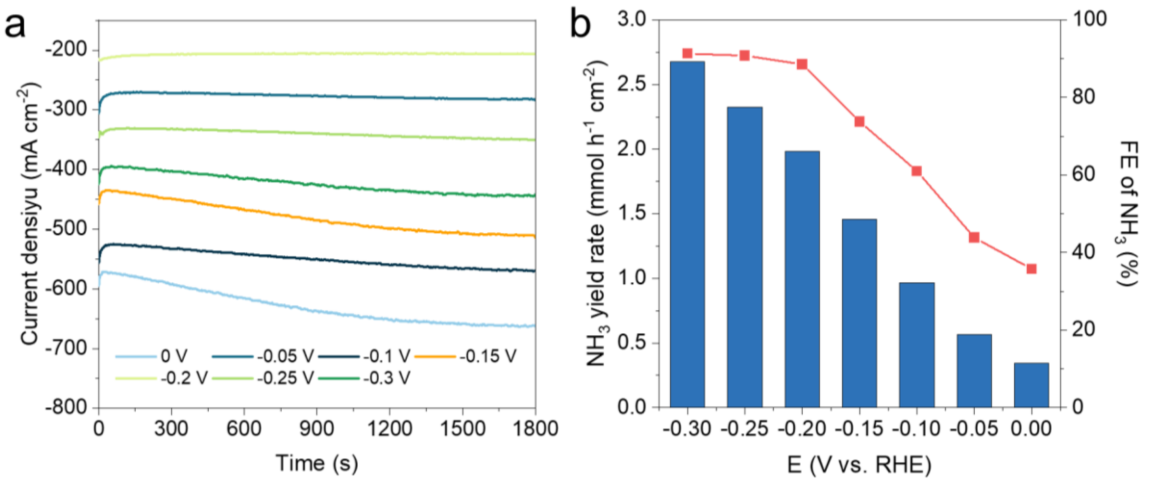

Supplementary Figure 25 | Nitrate reduction performance of $Ni(OH)_x$/CuO electrode. a. Potential-dependent i-t curves. b. Corresponding NH_3 yield rate and FE of NH_3.

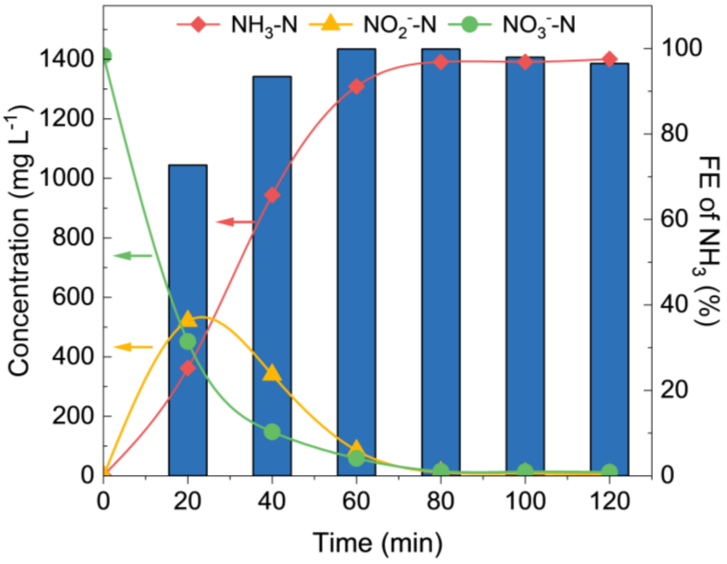

Supplementary Figure 26 | Complete nitrate conversion using Ni(OH)$_x$/Cu with an initial 1 M KOH with 0.1 M NO_3^- (equals to 1400 mg L^{-1} NO_3^-−N) at −0.25 V vs. RHE.

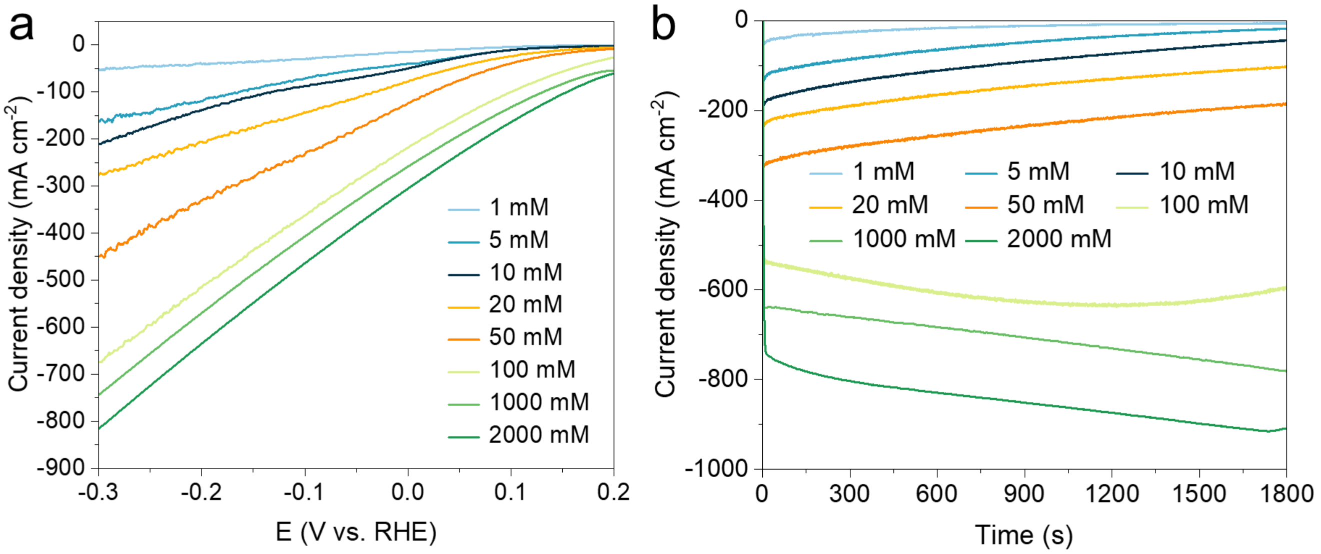

Supplementary Figure 27 | Evaluation of the performance of Ni(OH)$_x$/Cu in electrolytes with different NO_3^- concentrations. a. LSV curves of Ni(OH)$_x$/Cu in 1 M KOH containing different concentrations of NO_3^- at a scan rate of 5 mV s^{-1}. **b.** I-t curves of Ni(OH)$_x$/Cu under different concentrations of NO_3^- at −0.25 V (vs. RHE).

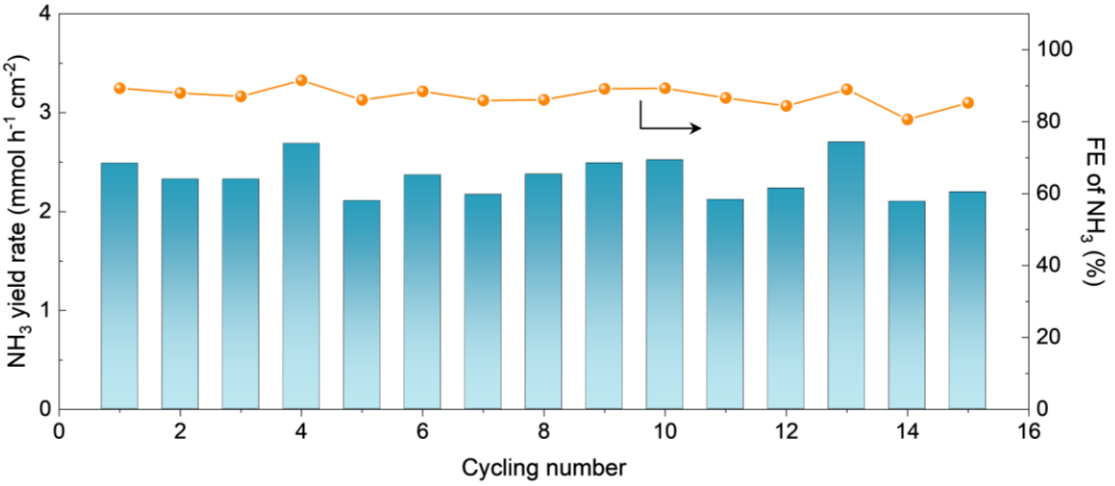

Supplementary Figure 28 | NH_3 yield rate and corresponding NH_3 FE over the 15 independent tests using $Ni(OH)_x/Cu$ electrode at −0.25 V (vs. RHE) in 1 M KOH with 0.1 M NO_3^-.

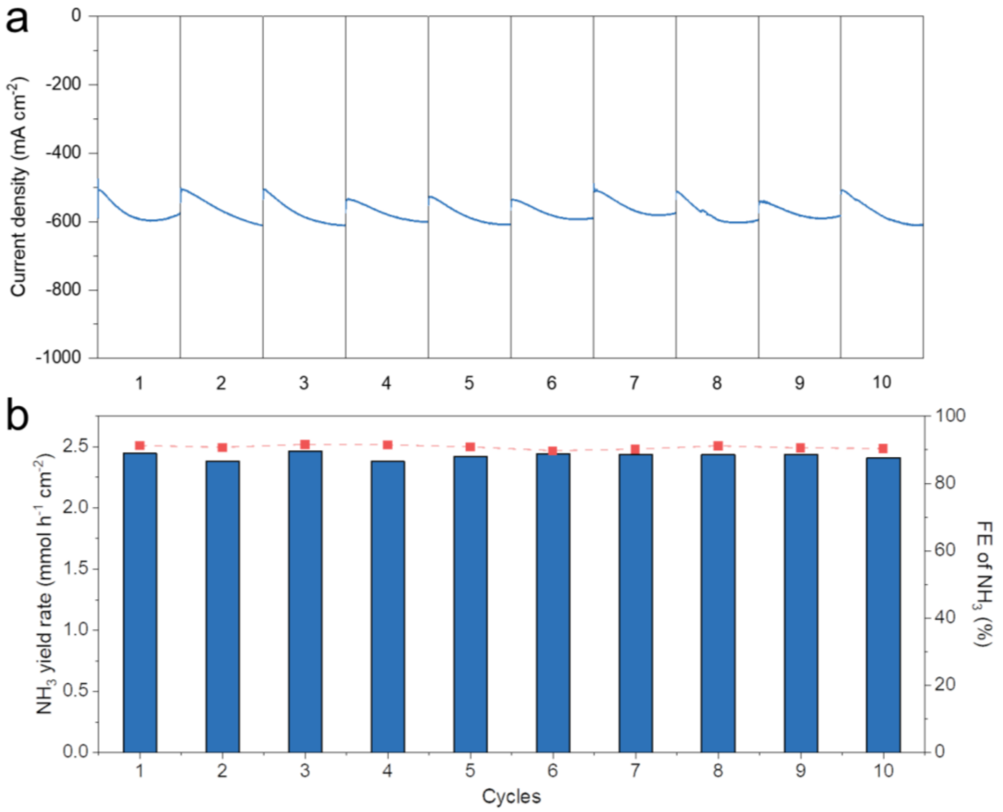

Supplementary Figure 29 | 10 cyclic test of the $Ni(OH)_x/Cu$ electrode. a. I-t curves. **b.** Corresponding NH_3 yield rate and FE of NH_3.

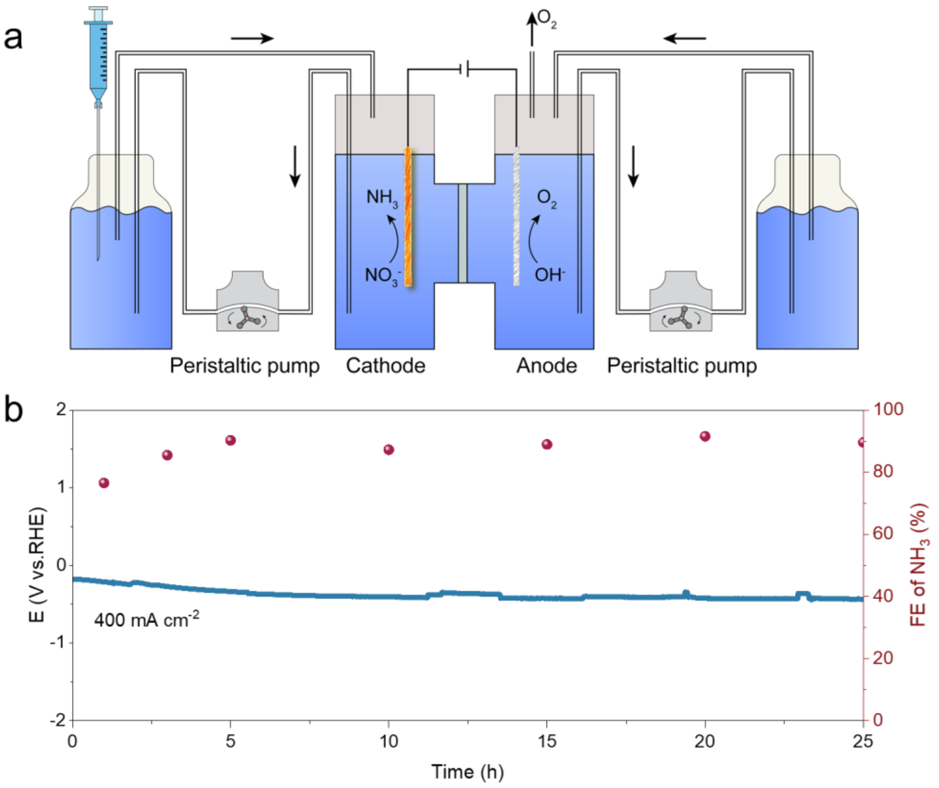

Supplementary Figure 30 | Long-term stability test in H-type flow cell. a. Schematic of the H-type flow cell. **b.** Long-term chronopotentiometry stability test of $Ni(OH)_x/Cu$ at 400 mA cm^{-2} with 0.1 M NO_3^- using the flow system.

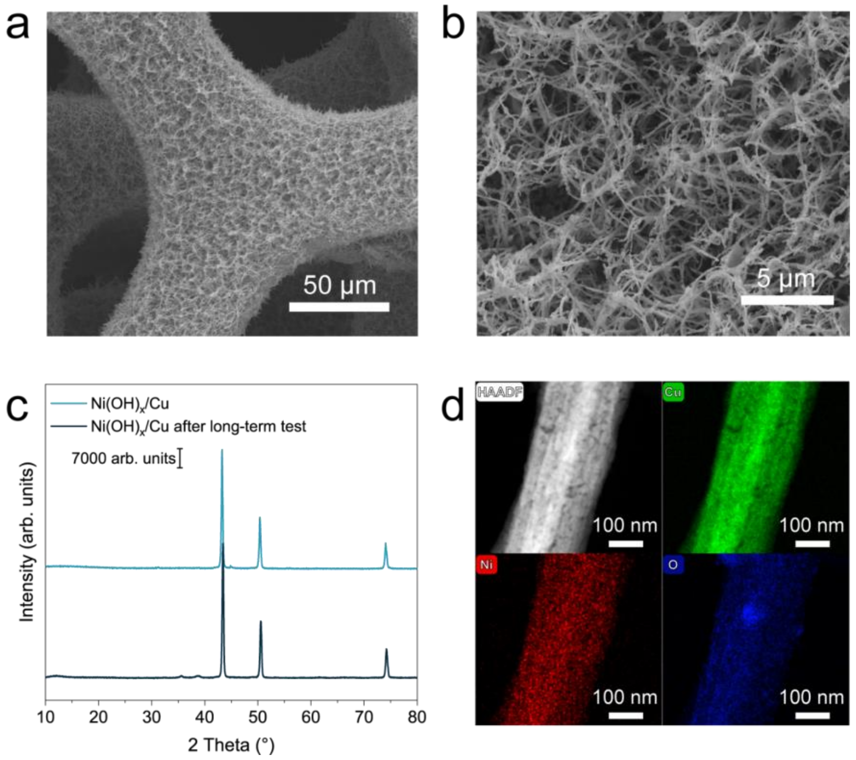

Supplementary Figure 31 | Structure characterization of the tested $Ni(OH)_x/Cu$ sample. a-b. SEM images of $Ni(OH)_x/Cu$ electrode after 100 h of electrocatalysis. **c.** Comparison of XRD patterns before and after long-term test. **d.** HAADF-STEM image and corresponding EDX images.

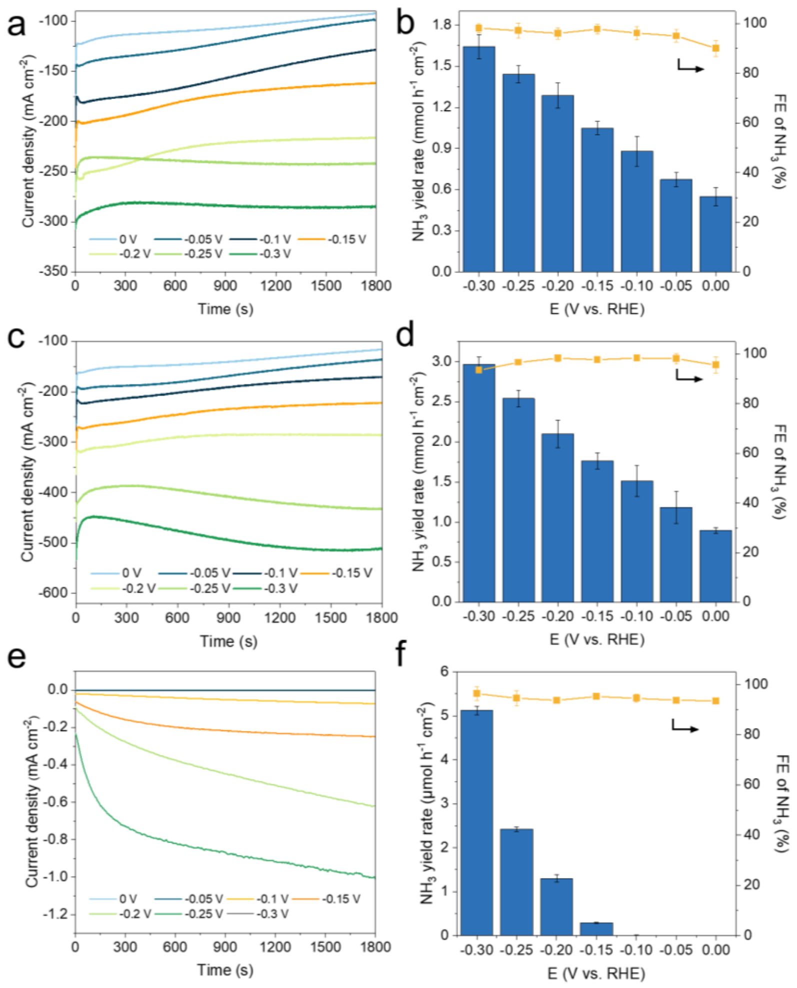

Supplementary Figure 32 | eNO_2^-RR performance evaluation in 1 M KOH with 0.1 M KNO_2. a, c, e. I-t curves of Cu (a), $Ni(OH)_x$/Cu (c), and $Ni(OH)_2$ (e). **b, d, f.** Corresponding NH_3 yield rate and FE of NH_3 of Cu (b), $Ni(OH)_x$/Cu (d), and $Ni(OH)_2$ (f). The error bars represent the standard deviation from at least three independent measurements.

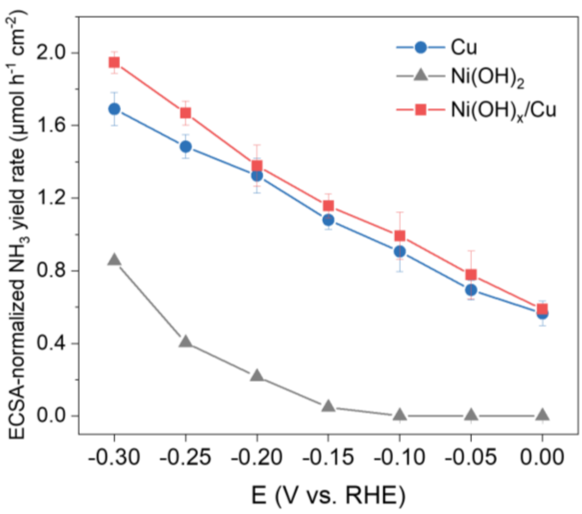

Supplementary Figure 33 | ECSA-normalized NH_3 yield rate of Cu, $Ni(OH)_x$/Cu, and $Ni(OH)_2$ in eNO_2^-RR. The error bars represent the standard deviation from at least three independent measurements.

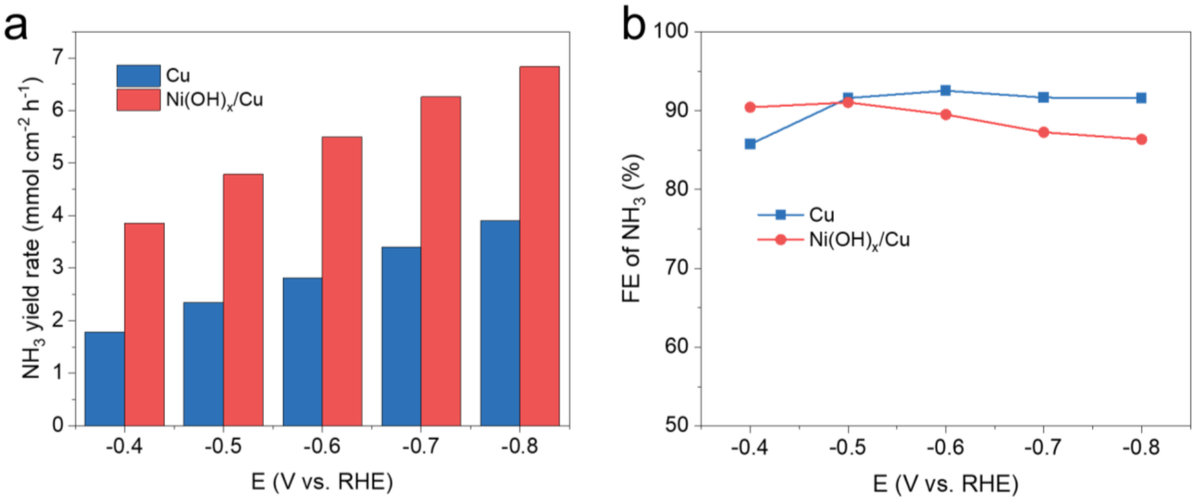

Supplementary Figure 34 | eNO$_3^-$RR performance at more negative potentials. a. NH_3 yield rate of Cu and $Ni(OH)_x/Cu$. **b.** The corresponding FE of NH_3.

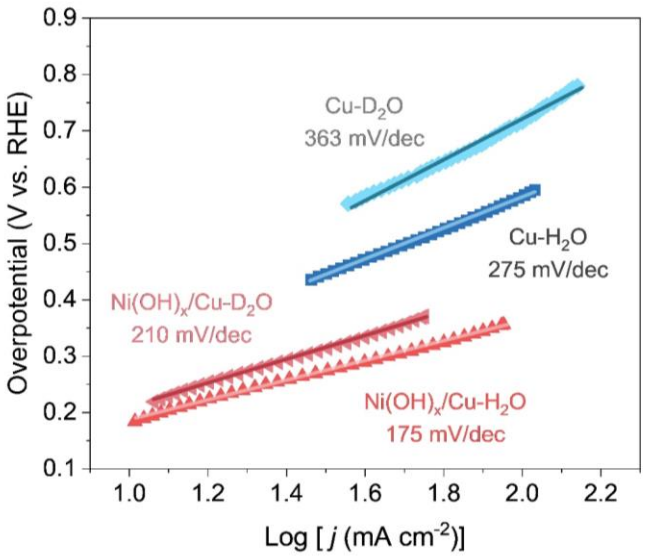

Supplementary Figure 35 | Tafel slopes of HER and DER over Cu and $Ni(OH)_x/Cu$. The Tafel slopes of Cu and $Ni(OH)_x/Cu$ are both larger than 120 mV/dec, indicating that the Volmer step (water dissociation) is the rate-determining step in HER. The water dissociation kinetic over $Ni(OH)_x/Cu$ is faster than that of Cu because of its smaller Tafel slope. In addition, after replacing H_2O with D_2O, the change in Tafel slope over $Ni(OH)_x$.Cu is smaller than that over Cu, suggesting a more efficient water dissociation kinetic over Ni(OH)x/Cu.

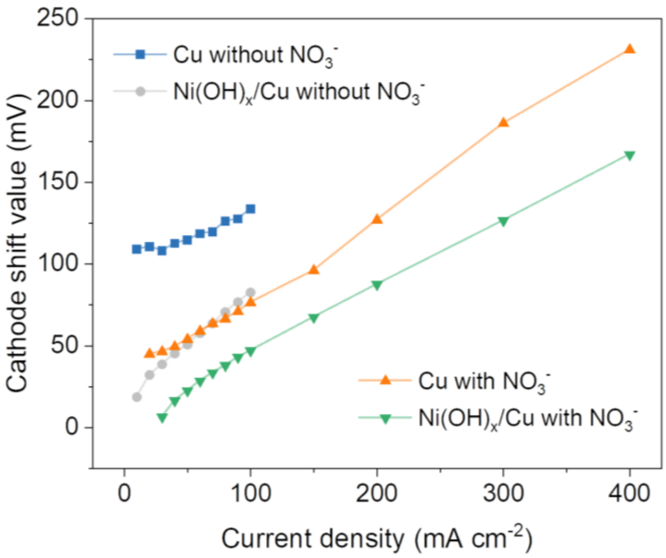

Supplementary Figure 36 | Cathode shift values under different current densities over Cu and Ni(OH)$_x$/Cu electrodes in the presence/absence of NO_3^-.

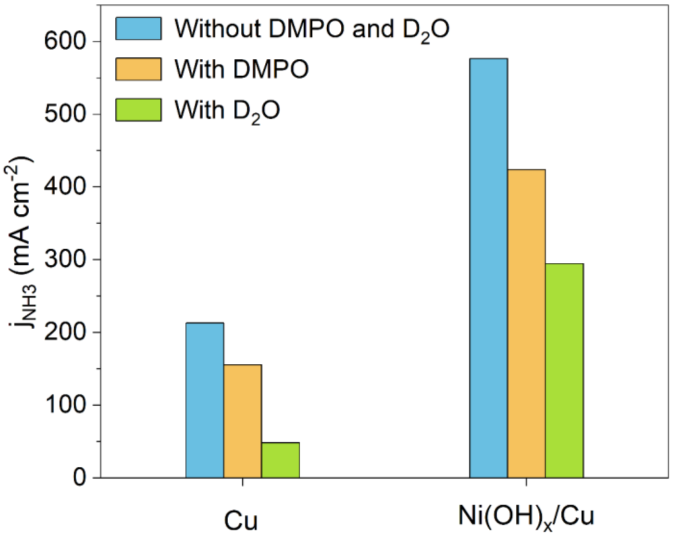

Supplementary Figure 37 | NH_3 partial current density with the addition of DMPO or D_2O over Cu and Ni(OH)$_x$/Cu at −0.25 V (vs. RHE).

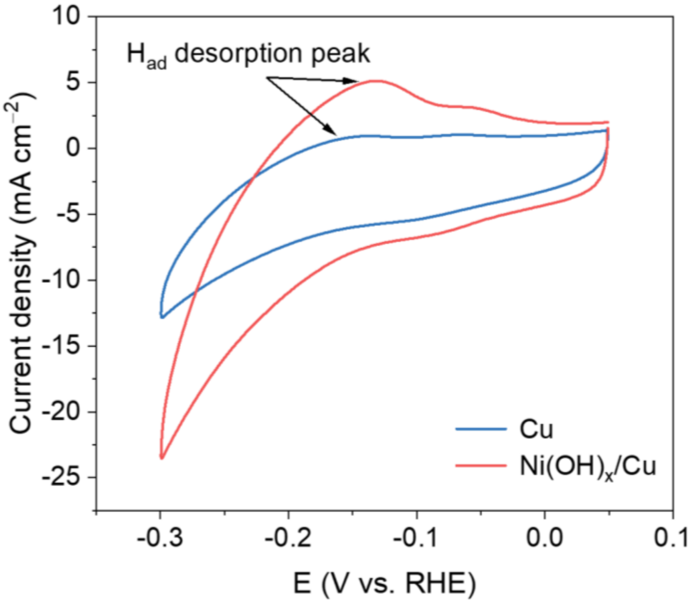

Supplementary Figure 38 | CV curves in Ar-saturated 1 M KOH with a scan rate of 100 mV s^{-1}. The CV curves manifest two regions: adsorption/desorption peaks of underpotentially deposited hydrogen (H_{upd}) at the potential range of −0.2-−0.1 V and a double-layer potential range of −0.1-0.05 V. The H_{ad} desorption peak of Ni(OH)$_x$/Cu at the H_{upd} range is more significant than that of Cu, suggesting the existence of more H_{ad} on the surface of Ni(OH)$_x$/Cu.

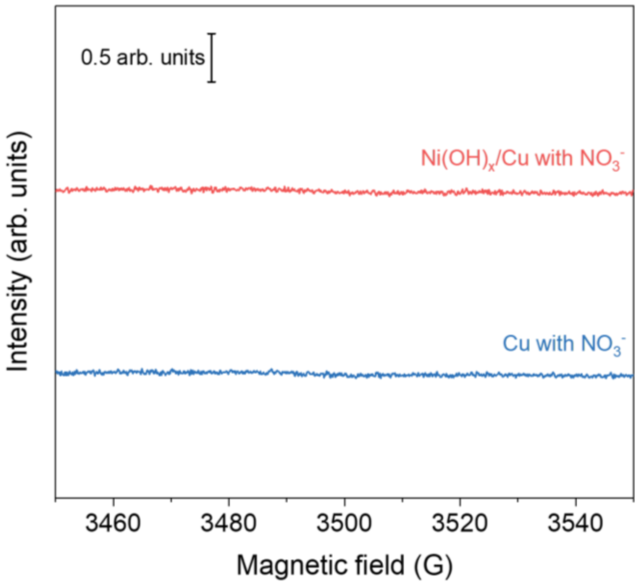

Supplementary Figure 39 | DMPO-involved EPR spectra of the solutions obtained after 10 min of eNO_3^-RR test over Cu and Ni(OH)$_x$/Cu.

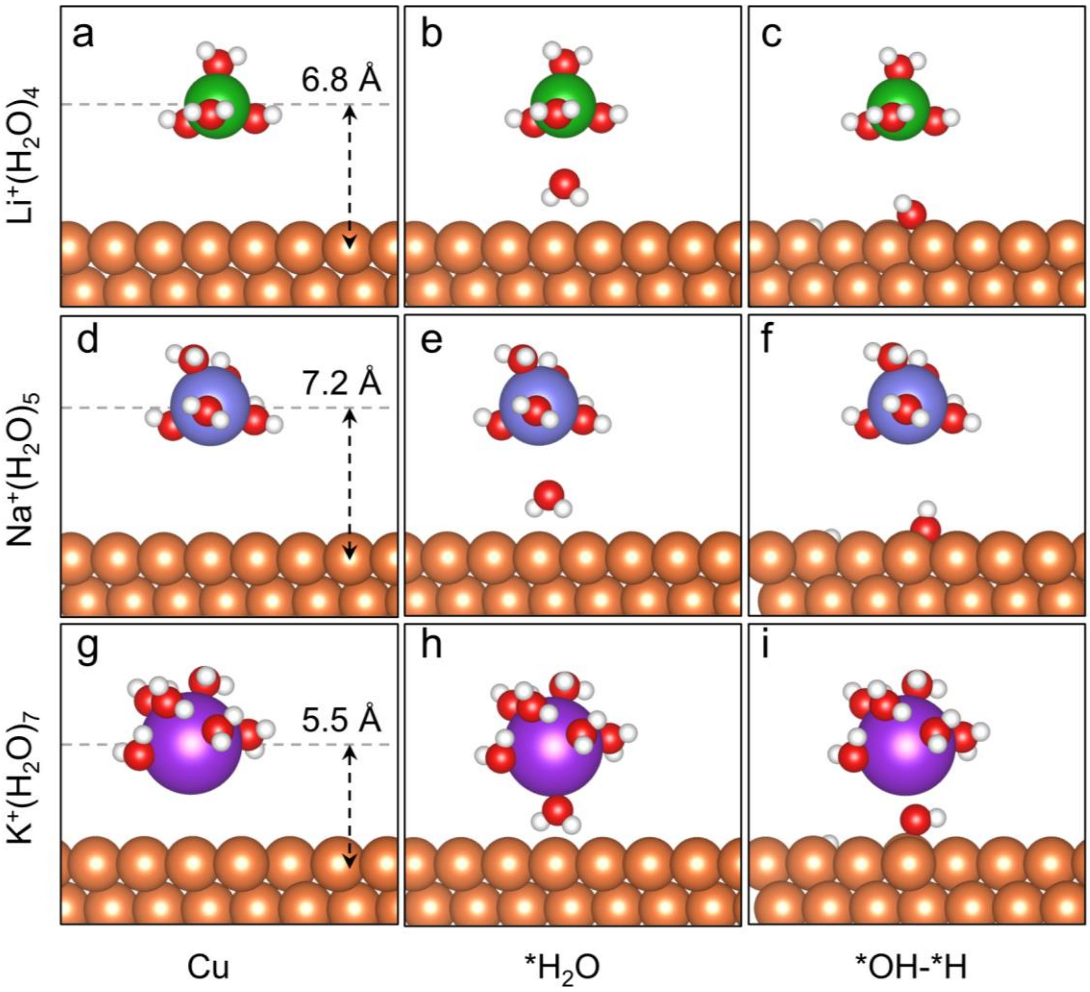

Supplementary Figure 40 | Atomic configurations of water adsorption and dissociation on Cu in the presence of different cation hydrates. a-c. $Li^+(H_2O)_4$**. d-f.** $Na^+(H_2O)_5$. **g-i.** $K^+(H_2O)_7$.

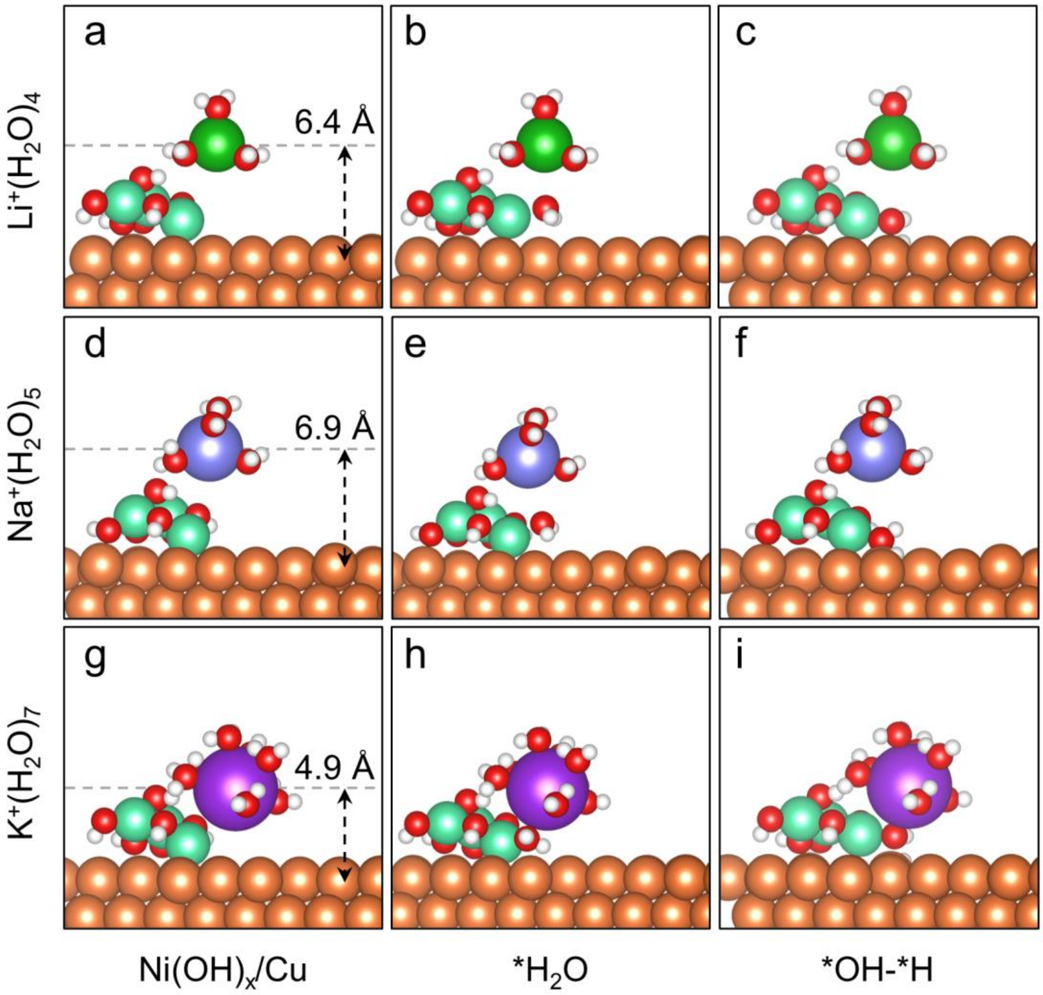

Supplementary Figure 41 | Atomic configurations of water adsorption and dissociation on $Ni(OH)_x$/Cu in the presence of different cation hydrates. a-c. $Li^+(H_2O)_4$**. d-f.** $Na^+(H_2O)_5$. **g-i.** $K^+(H_2O)_7$.

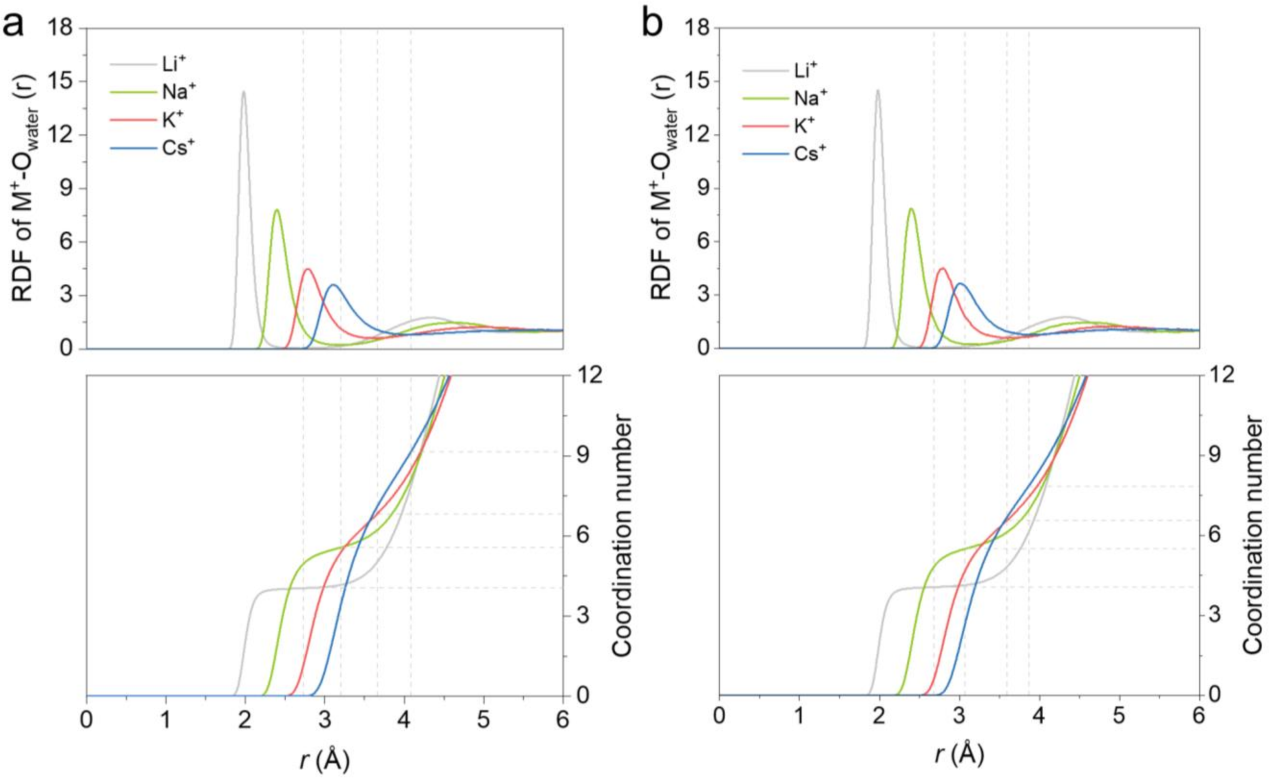

Supplementary Figure 42 | Cation−water coordination number determination. a-b. Radial distribution function (RDF) profiles of Li^+-, Na^+-, K^+- and Cs^+-O (in H_2O) on Cu (a) and $Ni(OH)_x$/Cu (b), as well as cation−water coordination number as function of radial distance.

The pronounced first peak and deep minimum indicate a highly structured first solvation shell. The integrated quantities of these first peaks are used to calculate the average coordination numbers of water molecule around cations. Integration up to the first minimum of the cation–O RDF gives the coordination numbers of 4.04, 5.49, 6.86, and 9.17 for Li^+, Na^+, K^+, and Cs^+, respectively, when using Cu as the substrate. While these coordination numbers become 4.06, 5.48, 6.57, and 7.87 over $Ni(OH)_x$/Cu.

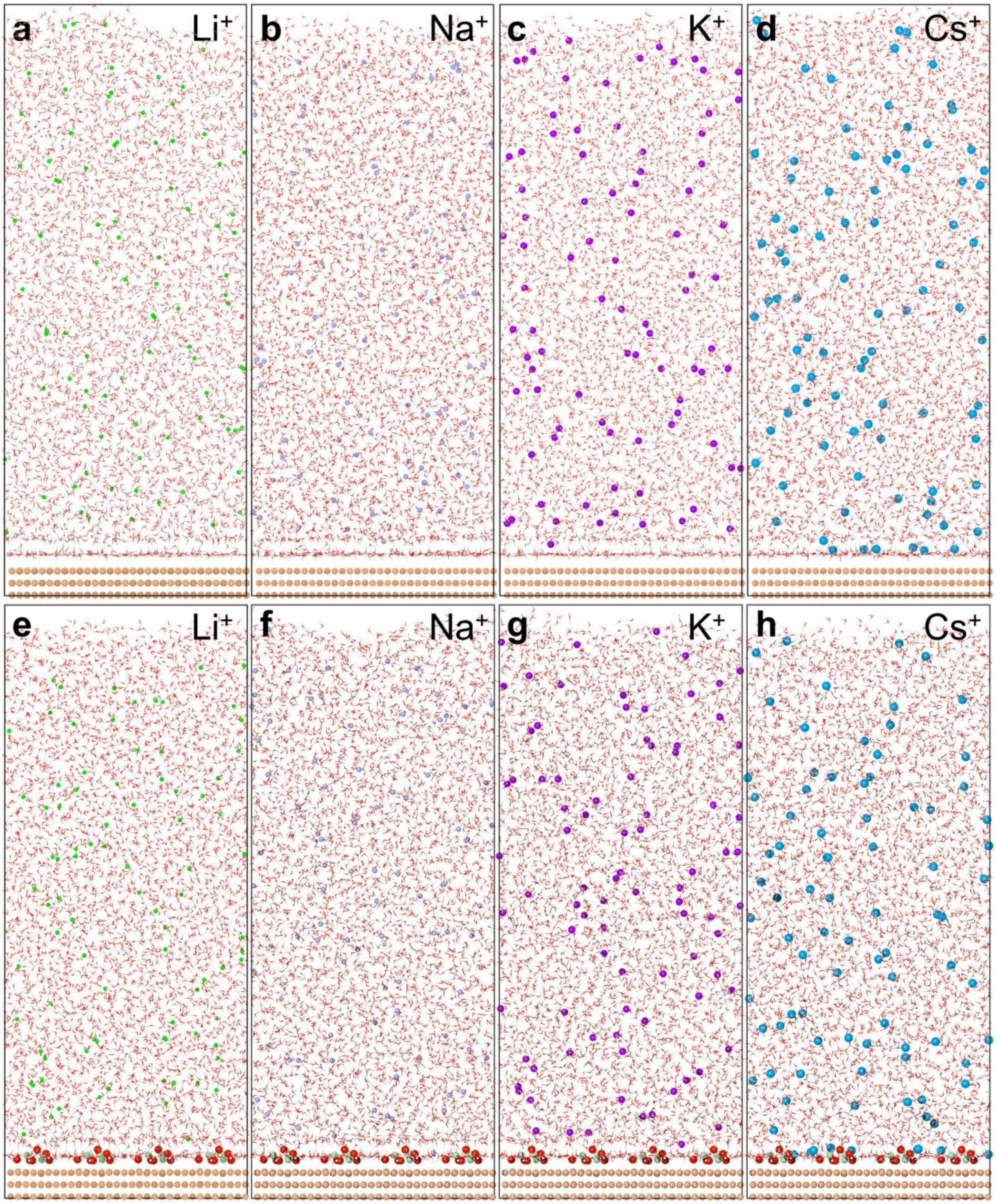

Supplementary Figure 43 | Snapshots of MD simulation. Spatial distribution of Li^+, Na^+, K^+, and Cs^+ on Cu (a-d) and $Ni(OH)_x$/Cu (e-h).

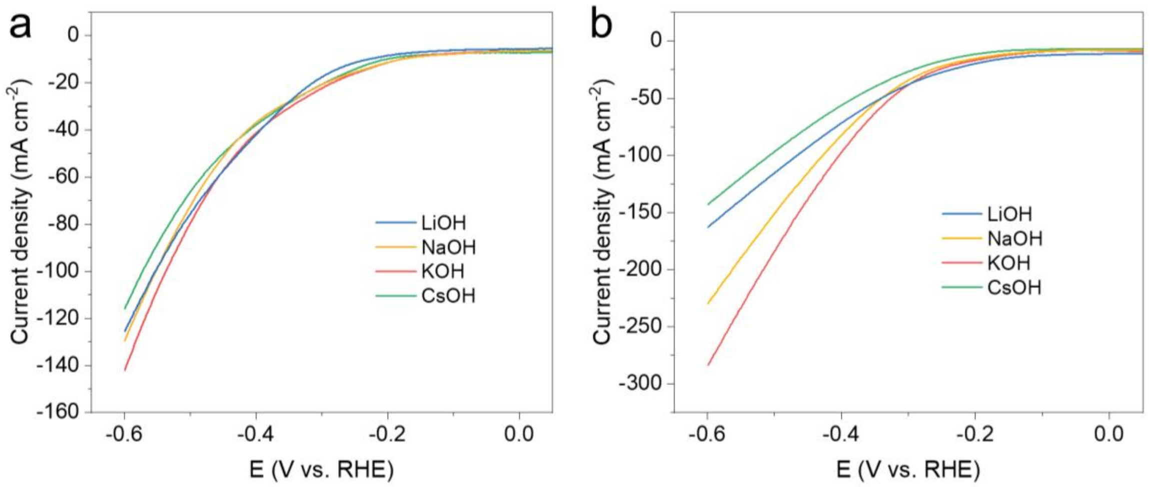

Supplementary Figure 44 | HER performance evaluation in the presence of different alkali metal cations. a. Cu electrode. **b.** $Ni(OH)_x$/Cu electrode.

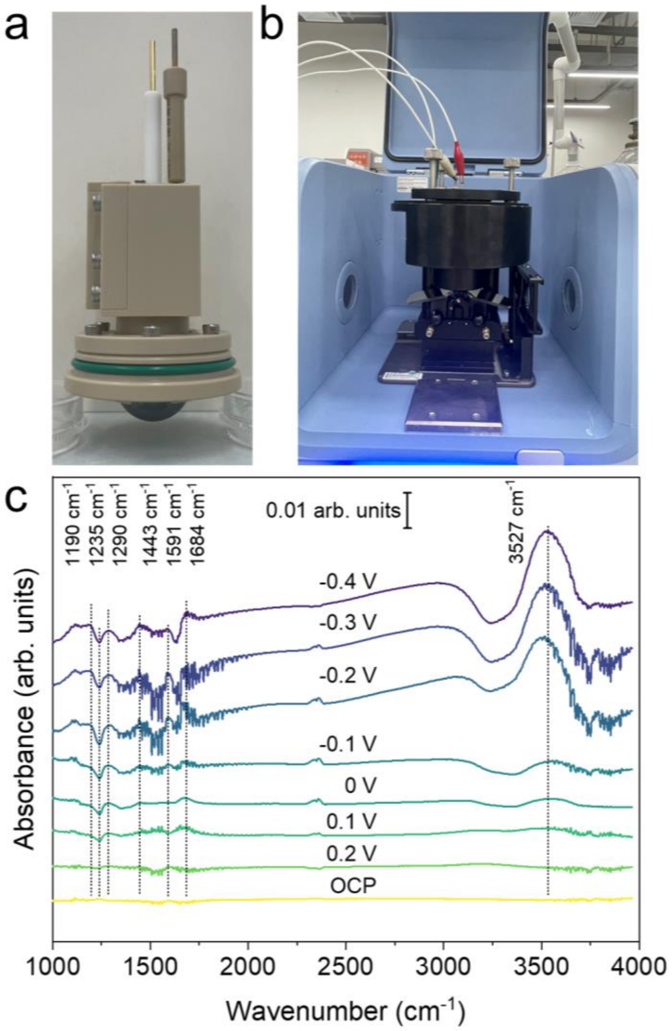

Supplementary Figure 45 | *In situ* electrochemical ATR-SEIRAS. a. Photograph of the home-made *in situ* ATR-SEIRAS cell. **b.** The experimental set-up for *in situ* ATR-SEIRAS test. **c.** *In situ* ATR-SEIRAS spectra of nitrate reduction over $Ni(OH)_x/Cu$.

The infrared absorption signals were collected from 1000 cm^{-1} to 4000 cm^{-1} during a negative scan from 0.2 V to −0.4 V. A negative band showed up at 1235 cm^{-1} corresponding to the consumption of NO_3^-. The peak that appeared at 1190 cm^{-1} was characteristic of the production of hydroxylamine (NH_2OH). The band at 1290 cm^{-1} was assignable to the wagging mode of -NH_2. The emergence of -H-N-H bending mode and N-H stretching mode was observed at 1443 cm^{-1} and 3527 cm^{-1}, suggesting a hydrogenation process toward NH_3 formation. NO was also a common intermediate during nitrate reduction, and its FTIR peak was centered at 1600-1690 cm^{-1}. However, the O-H bending of water at 1623cm^{-1} would interfere with the identification of the NO intermediate.

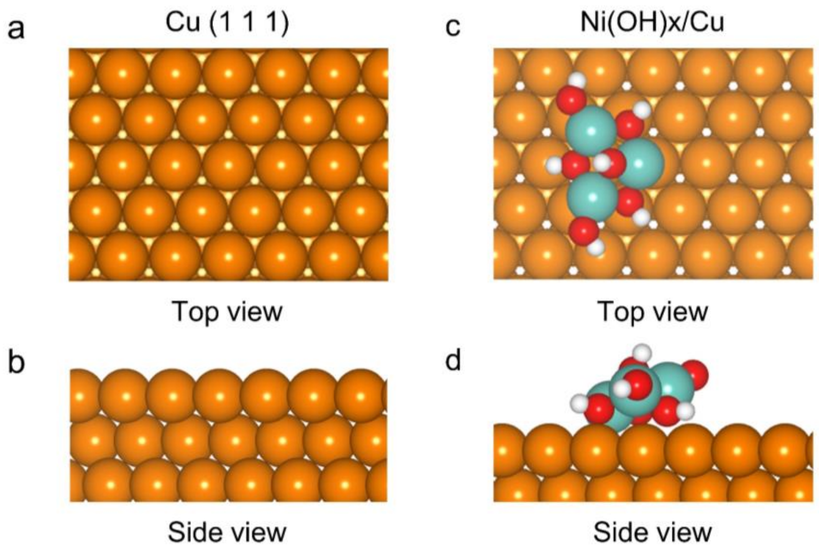

Supplementary Figure 46 | Geometries of the molecular structures for DFT calculations. a-b. Top view and side view of Cu (1 1 1) facet structural model. **c-d.** Top view and side view of $Ni(OH)_x/Cu$ structural model.

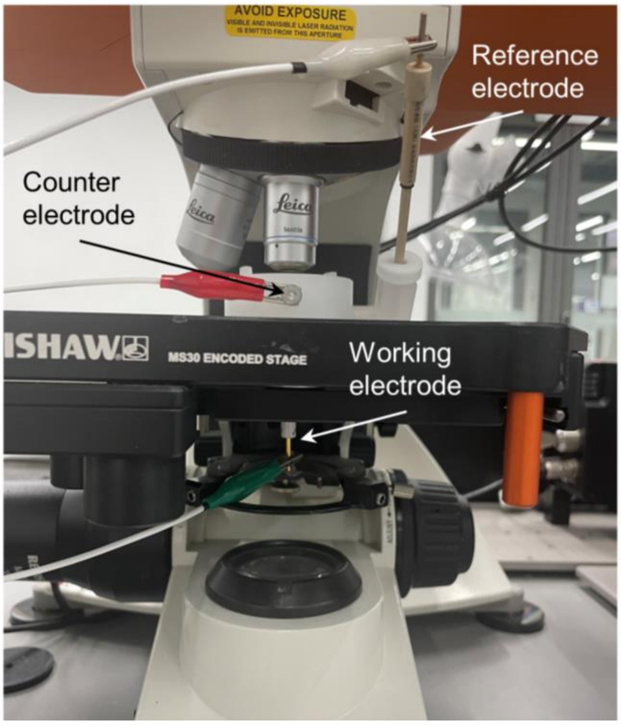

Supplementary Figure 47 | Photograph of the experimental set-up for *in situ* Raman measurement. For testing *in situ* Raman spectra, $Ni(OH)_x/Cu$ and Cu nanowires were ultrasonically collected from the copper foam. The catalyst ink was drop-casted on the glassy carbon electrode (working electrode). A Pt wire and a Hg/HgO electrode were used as counter electrode and reference electrode, respectively.

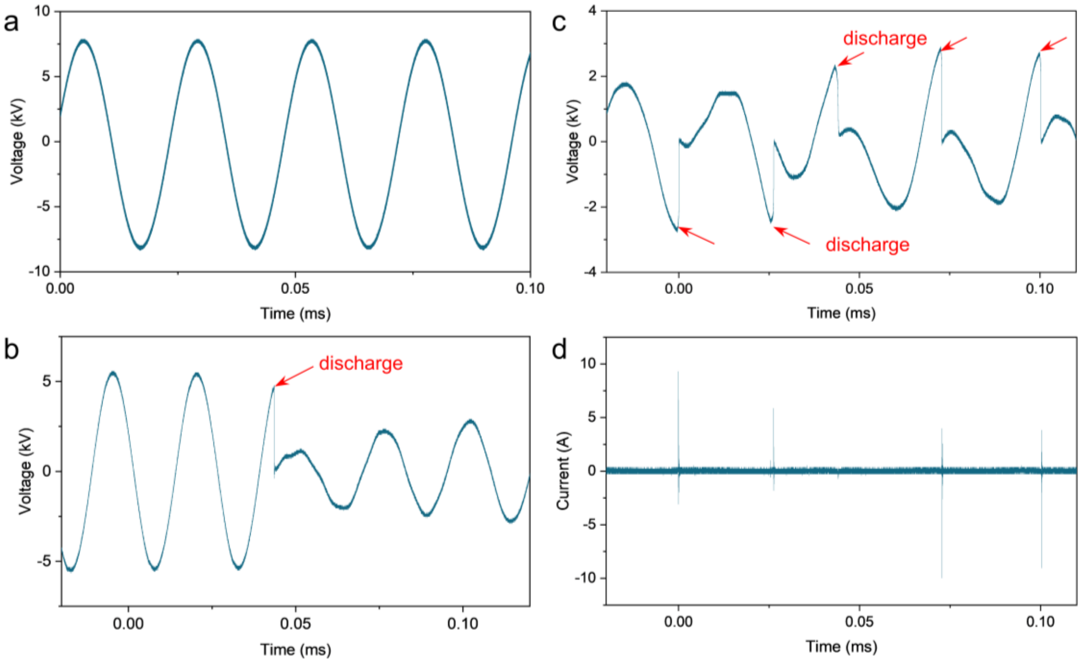

Supplementary Figure 48 | Parameters of the spark discharge NTP. a. The voltage output of the neon-sign transformer at 220 V (AC). **b.** The initial breakdown voltage of the spark discharge. **c-d.** The working voltage and current of the spark discharge.

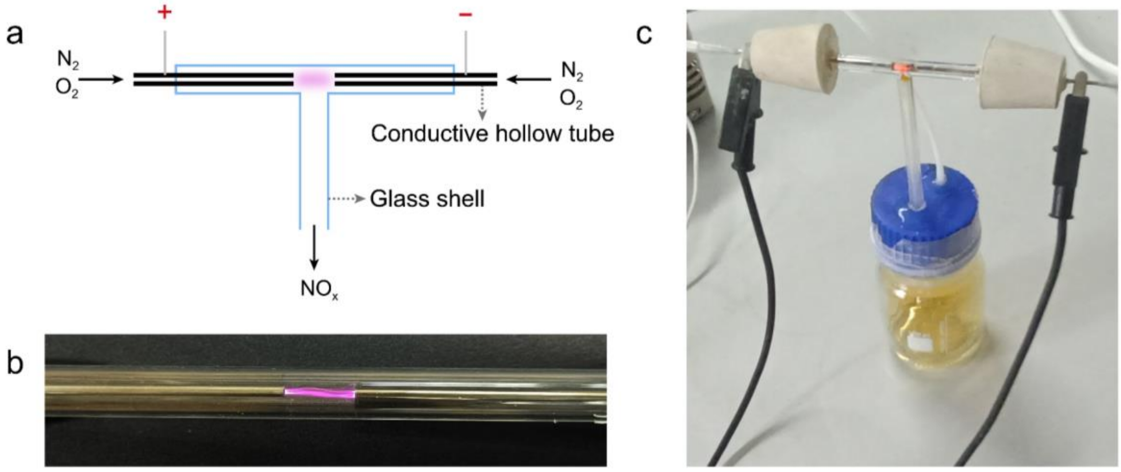

Supplementary Figure 49 | The spark discharge NTP configuration. a. Schematic of the configuration of spark discharge NTP. **b.** Photograph of the spark discharge NTP. **c.** Color change of the gas after passing through the spark discharge NTP.

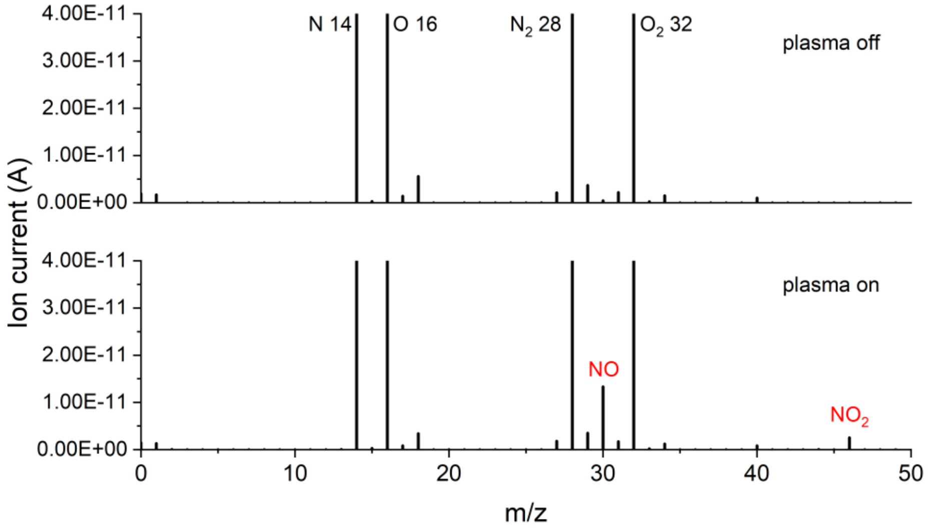

Supplementary Figure 50 | Mass spectra of the feeding gas with or without spark discharge NTP. The signals of NO and NO_2 are barely observable when the spark discharges non-thermal plasma is off. After turning on the plasma, the signals of NO (*m*/*z* = 30) and NO_2 (*m*/*z* = 46) appear, indicating that the NO and NO_2 species are generated during spark discharge.

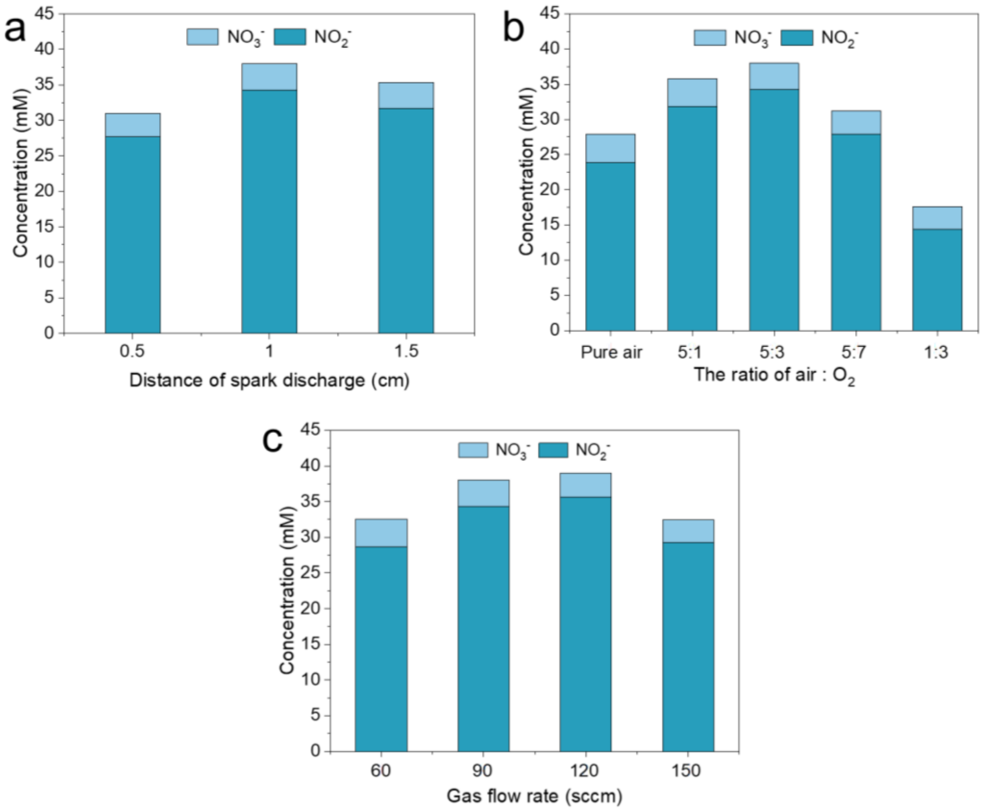

Supplementary Figure 51 | Effect of different spark discharge NTP conditions on NO_x^- concentration in 25 mL of 1 M KOH absorbing solution for 10 min of operation. a. Distance of spark discharge. **b.** The ratio of air:O_2 in the gas inlet. **c.** Total gas flow rate.

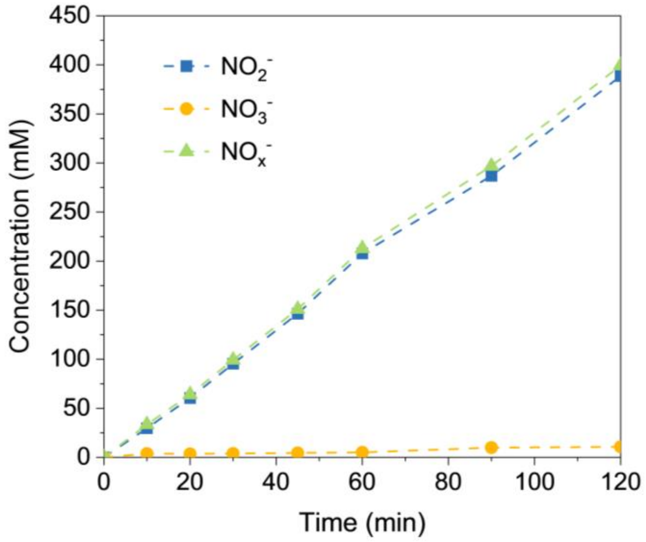

Supplementary Figure 52 | Stability evaluation of the spark discharge NTP. Concentrations of NO_2^-, NO_3^-, and total NO_x^- in 25 mL of 1 M KOH solution under different discharge time.

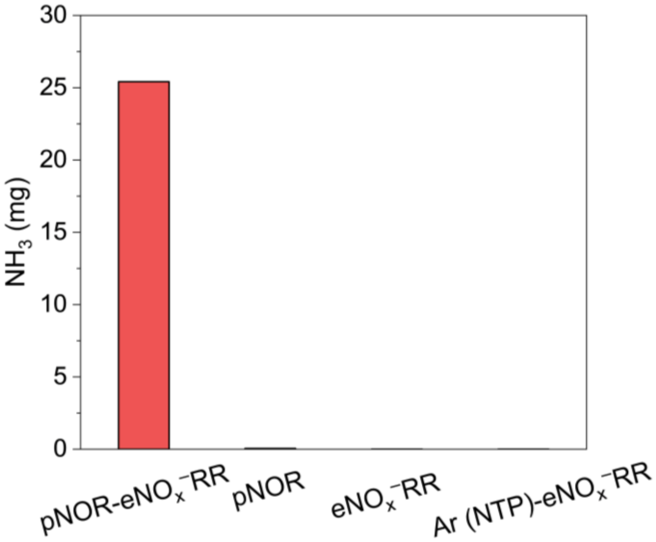

Supplementary Figure 53 | Comparison of NH_3 yield under different operation models. Ar was used as feeding gas instead of air in the Ar(NTP)-eNO_x^-RR experiment.

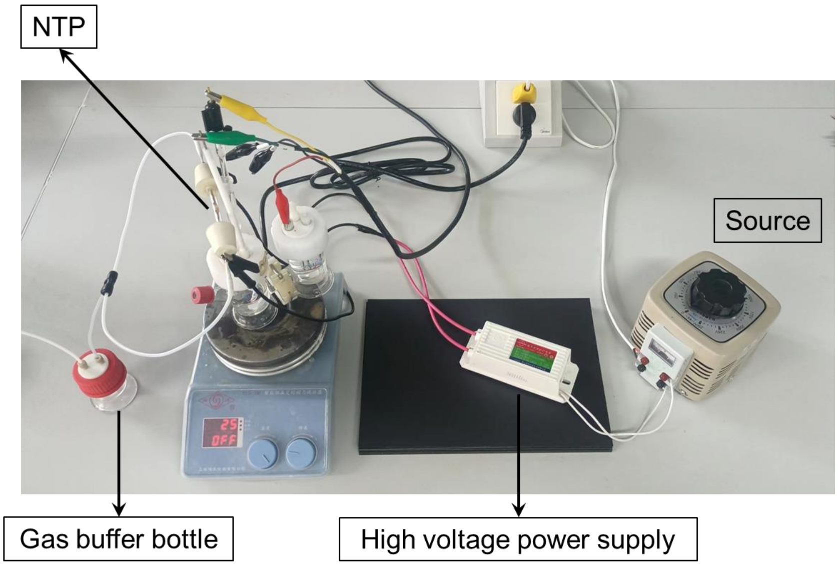

Supplementary Figure 54 | Configuration of the pNOR-eNO_x^-RR system for batch experiments. In the batch experiments, gas reactants are purged to the spark discharge plasma after mixing in the gas buffer bottle. The spark discharge plasma is generated by a high voltage power supply, which connects a manual contacting voltage regulator.

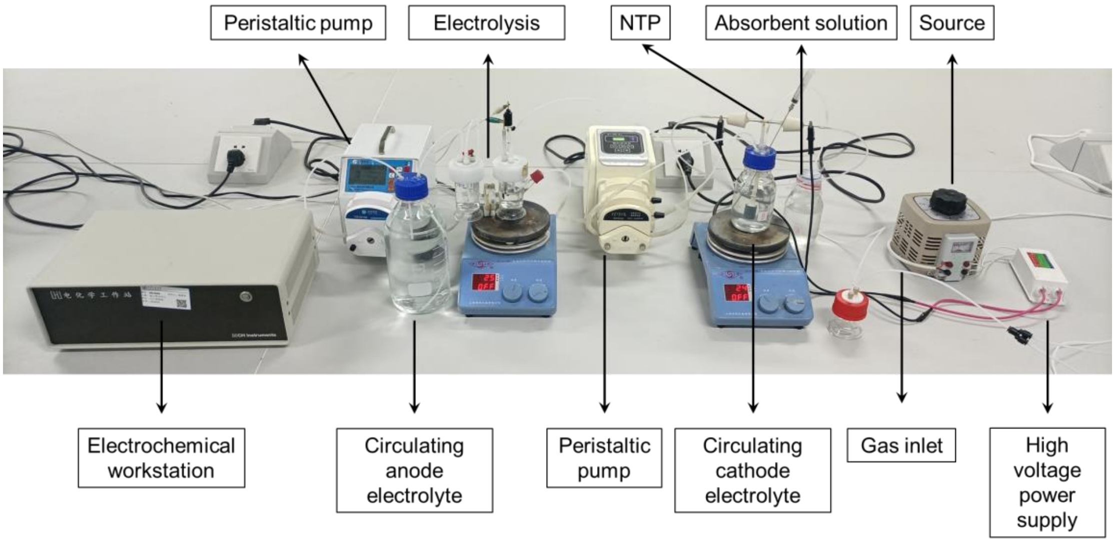

Supplementary Figure 55 | Configuration of the pNOR-eNO_x^-RR system for long-term continuous operation. Different from the set-up for batch experiments, peristaltic pumps are used to make the electrolyte in anode and cathode flow. The gas is purged in the circulating cathode electrolyte after the spark discharge plasma treatment. An acid solution is used to absorb NH_3 in the gas outlet.

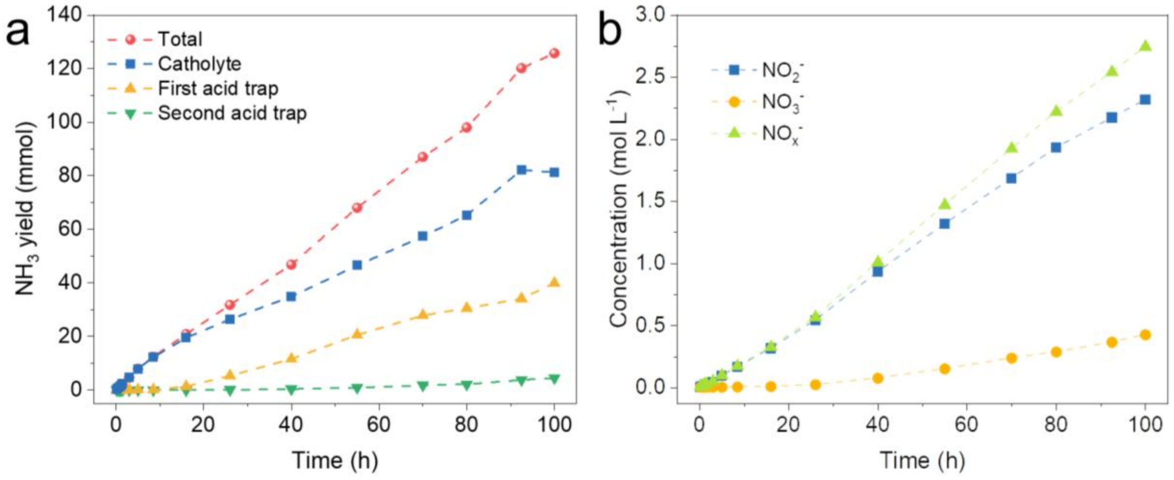

Supplementary Figure 56 | Concentration variations of NH_3 and NO_x^-. a. The distribution of produced NH_3. **b.** Concentration variations of NO_3^- and NO_2^- in the catholyte.

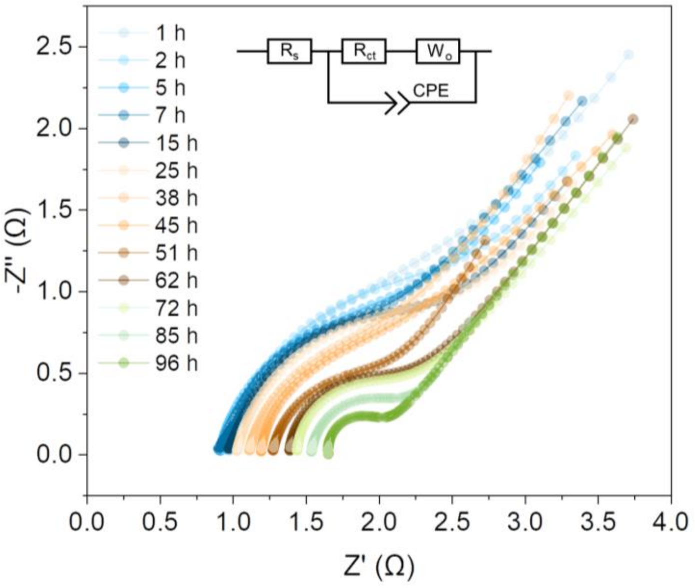

Supplementary Figure 57 | Electrochemical impedance spectroscopy (EIS) of $Ni(OH)_x/Cu$ obtained at different operation time.

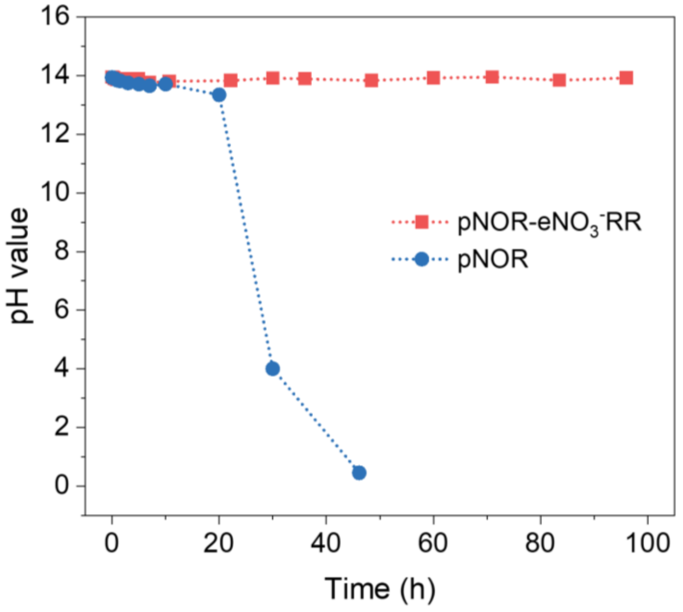

Supplementary Figure 58 | pH variations of the catholyte in pNOR-eNO$_x^-$RR tandem system and absorption solution of pNOR without eNO$_x^-$RR.

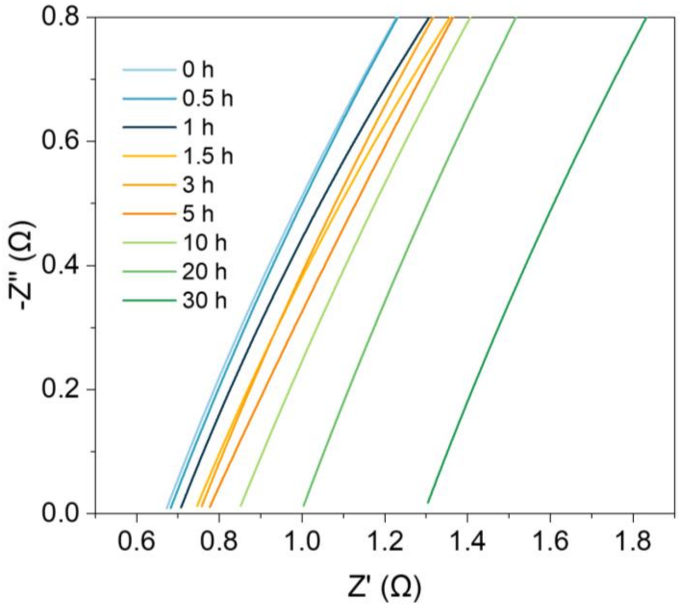

Supplementary Figure 59 | EIS obtained in the absorption solution of pNOR without eNO$_x^-$RR. The constantly generated NO and NO_2 species consume OH^-, not only leading to a gradual decrease in pH but also a continuous increase in solution resistance.

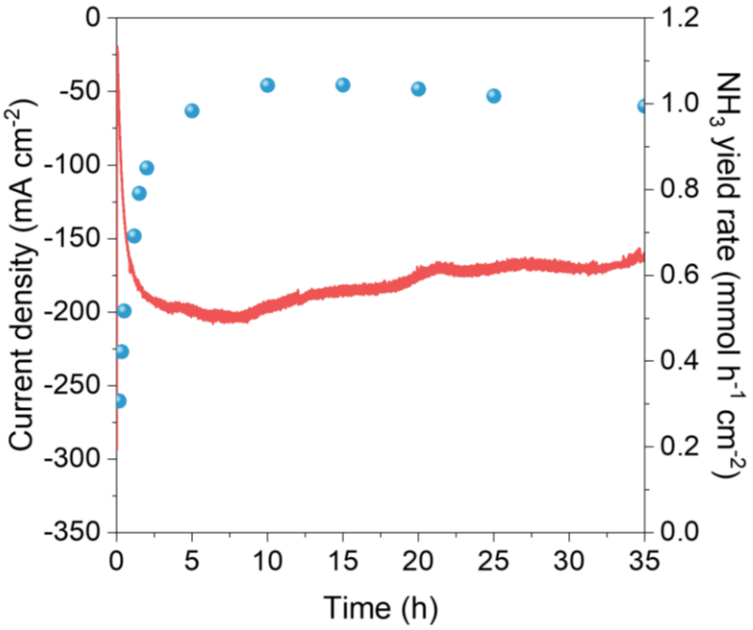

Supplementary Figure 60 | Long-term continuous test of pNOR-eNO$_x^-$RR system using Cu electrode. The Cu electrode can only deliver a current density of ca. 175 mA cm^{-2} and an NH_3 yield rate of ca. 1.0 mmol h^{-1} cm^{-2}. The performance of the Cu electrode is inferior to that of the $Ni(OH)_x$/Cu electrode, highlighting the important role of $Ni(OH)_x$ in conversing NO_x^- to NH_3 on the Cu surface.

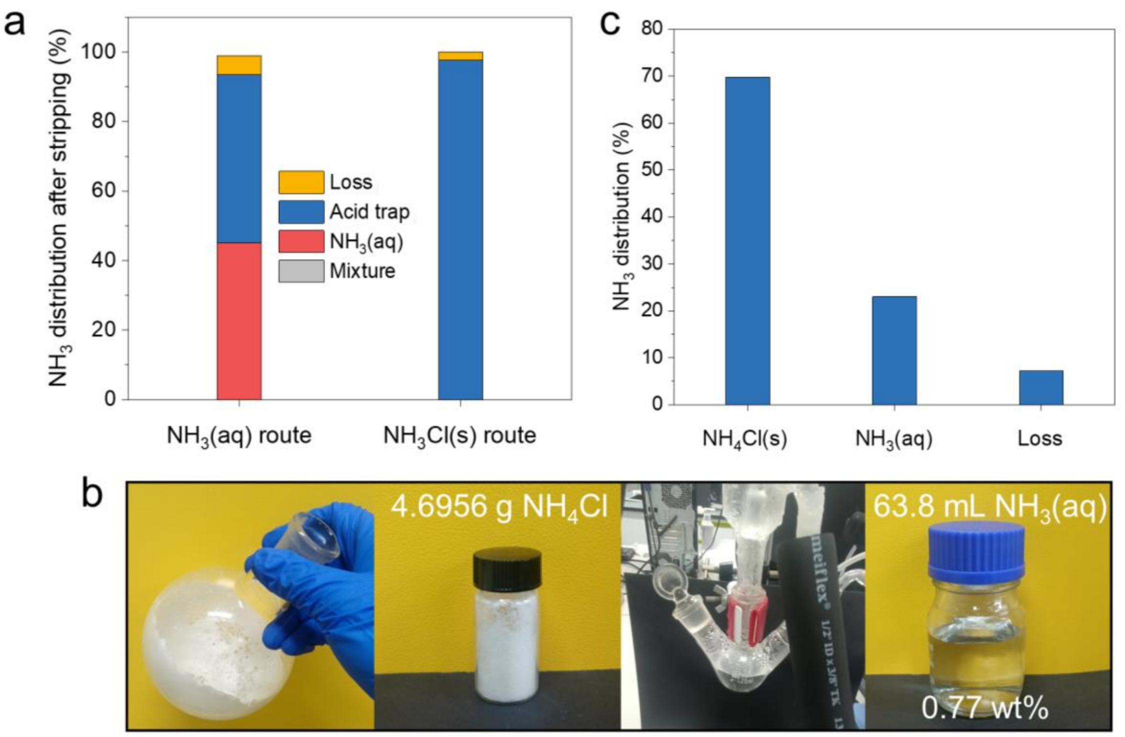

Supplementary Figure 61 | NH_3 products collection. a. NH_3 distribution after the air striping procedure. **b.** Photographs of collected NH_4Cl (s) after rotational evaporation and NH_3 (aq) after condensation. **c.** The percentages of NH_3 in the different products relative to the total amount of NH_3 obtained after the long-term test.

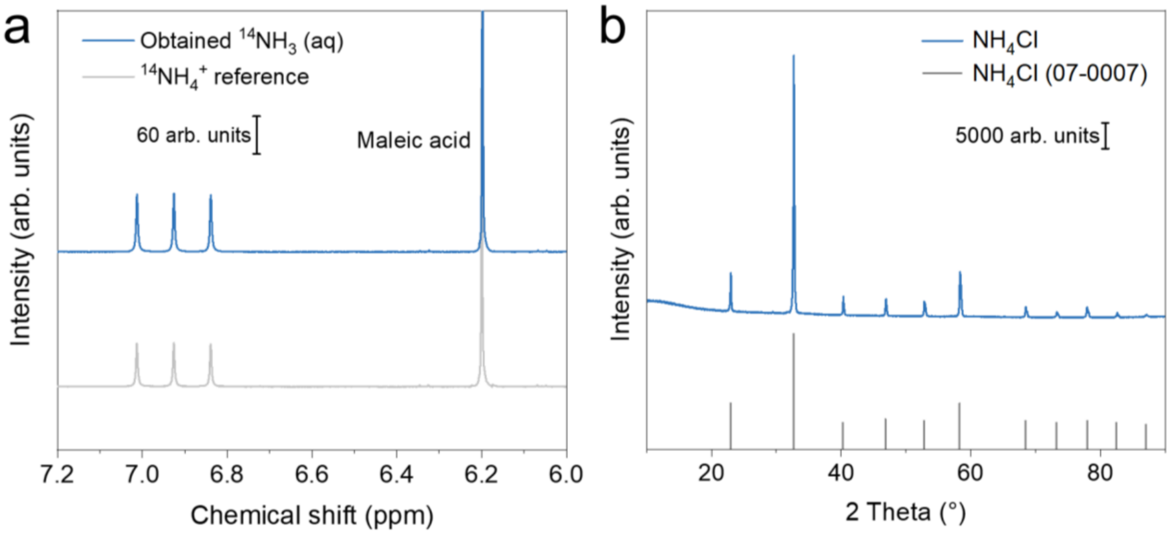

Supplementary Figure 62 | Ammonia products characterization. a. ^1H NMR spectra of the concentrated NH_3 aqueous product. **b.** XRD pattern of the solid NH_4Cl product.

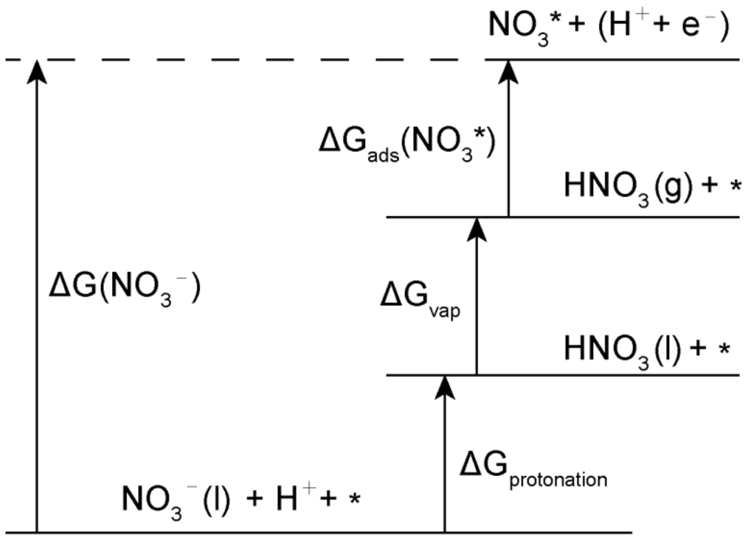

Supplementary Figure 63 | Thermodynamic cycle employed to calculate the overall free energy of NO_3^- adsorption.

Supplementary Table 1. EXAFS fitting parameters at the Ni *K*-edge for various samples.

Sample	Shell	*CN*[a]	*R*(Å)[b]	σ^2(Å^2)[c]	ΔE_0(eV)[d]	*R* factor
$Ni(OH)_2$	Ni-O	6*	2.045±0.001	0.0082±0.0027	1.7±0.7	0.0128
	Ni-Ni	6*	3.113±0.001	0.0096±0.0019	3.6±1.3	
Ni foil	Ni-Ni	12*	2.483±0.002	0.0061±0.0003	7.6±0.4	0.0023
$Ni(OH)_x/Cu$	Ni-O	4.6±0.3	2.050±0.001	0.0064±0.0012	-1.4±0.4	0.0042
	Ni-Cu	2.2±0.3	2.585±0.001	0.0085±0.0014	1.1±0.7	
	Ni-Ni	4.8±0.7	3.119±0.007			

[a]*CN*, coordination number; [b]*R*, the distance to the neighboring atom; [c]σ^2, the Mean Square Relative Displacement (MSRD); [d]ΔE_0, inner potential correction; *R* factor indicates the goodness of the fit. $S0^2$ was fixed to 0.954, according to the experimental EXAFS fit of $Ni(OH)_2$ by fixing *CN* as the known crystallographic value. * This value was fixed during EXAFS fitting, based on the known structure of Fe. Fitting range: $2.5 \leq k$ (/Å) ≤ 10.0 and $1.2 \leq R$ (Å) ≤ 3.2 ($Ni(OH)_2$); $2.5 \leq k$ (/Å) ≤ 12.0 and $1.0 \leq R$ (Å) ≤ 3.0 (Ni foil); $2.5 \leq k$ (/Å) ≤ 11.0 and $1.0 \leq R$ (Å) ≤ 3.5 ($Ni(OH)_x/Cu$). A reasonable range of EXAFS fitting parameters: $0.700 < S_0^2 < 1.000$; $CN > 0$; $\sigma^2 > 0$ Å^2; $|\Delta E_0| < 15$ eV; *R* factor < 0.02.

Supplementary Table 2. ICP-MS data for the $Ni(OH)_x/Cu$ nanowires that ultrasonic peeling from the Cu skeleton and $Ni(OH)_x/Cu$ bulk electrodes with different deposition times. The value of x in $Ni(OH)_x$ (ca. 1.53) was obtained from the result of EXAFS fitting.

Samples	Deposition time / min	Measured mass of Ni / mg	Measured mass of Cu / mg	$Ni(OH)_x$ / Cu mass ratio / %
$Ni(OH)_x/Cu$ nanowire	15	0.0351	3.99	1.27
	30	0.0902	5.67	2.30
	45	0.1430	6.23	3.31
$Ni(OH)_x/Cu$ bulk electrode	15	0.0378	14.00	0.39
	30	0.0474	10.70	0.64
	45	0.0578	11.00	0.76

Supplementary Table 3. Comparison of NH_3 yield rate and Faradaic efficiency of this work with previously reported works covering pathways of pNOR-eNO_x^-RR, eNRR, and Li-mediated eNRR.

Pathway for NH_3 synthesis	Catalyst	NH_3 yield rate (nmol s^{-1} cm^{-2})	Faradaic efficiency (%)	Reference
pNOR-eNO_x^-RR	$Ni(OH)_x/Cu$	833.3	92	This work
	Cu nanoparticles	38.5	94.8	[1]
	Cobaloxime	10.1	89	[2]
	Cu nanowires	45.3	~100	[3]
	$Ni_3B@NiB_{2.74}$	55.1	~100	[4]
	Cu_2Pd/CBC	32.0	93.79	[5]
	Co SAs/N-C	49.0	62	[6]
Li-mediated eNRR	Mo foil	0.97	27.9	[7]
	HBTCu	46.0	13.3	[8]
	Stainless-steel cloth	24.1	39.5	[9]
	Stainless steel cloth	30.4	35.3	[10]
	Cu foil	7.88	15.2	[11]
	Cu disk	51.3	69	[12]
	Mo foil	5.4	78	[13]
	Ni wire	150	100	[14]
eNRR	B_4C nanosheet	0.0434	15.95	[15]
	VN nanoparticles	0.33	6	[16]
	Cu/PI	0.281	6.56	[17]
	Eex-COF/NC	0.205	45.43	[18]
	MXene/SSM	0.077	4.62	[19]
	MoO_{3-x}/MXene	0.156	22.3	[20]
	ECOF/BCP	4.69	54.5	[21]
	$FeReS_3$	0.75	43	[22]
	BiO_x ADCs	0.092	30	[23]

Supplementary Note 1. Energy efficiency calculation of pNOR-eNO_3^-RR.

Calculation of energy consumption for pNOR:

Total NO_x^- produced = NO_x^- left in catholyte + Generated NH_3 = 0.9058 mol + 0.1257 mol = 1.0315 mol

Energy consumption for pNOR = Total energy consumed by pNOR in 100 hours / Total NO_x^- produced = 3.45 kWh / 1.0315 mol =3.3446 kWh mol^{-1} NO_x^-

Calculation of energy consumption for eNO_x^-RR:

We assume the electrolysis time is 1 hour.

Energy consumption for eNO_x^-RR = Total current × Cell voltage × Time / NH_3 produced = 0.24 A × 9.2 V × 1 h / 1.2574 mmol = 1.756 kWh mol^{-1} NH_3

Total energy consumption = Energy consumption for pNOR + Energy consumption for eNO_x^-RR = 3.3446 kWh mol^{-1} NO_x^- + 1.756 kWh mol^{-1} NH_3 = 5.1006 kWh mol^{-1} = 18.3622 MJ mol^{-1}

Calculation of energy efficiency:

The reaction of the plasma-assisted nitrogen oxidation is described by

$$\frac{1}{2}N_2\,(g) + \frac{5}{4}O_2\,(g) + OH^-\,(aq) \rightarrow NO_3^-\,(aq) + \frac{1}{2}H_2O\,(l)$$

$$\Delta H^0 = -120.715\ kJ/mol\ of\ NO_3^-$$

$$\Delta G^0 = -72.689\ kJ/mol\ of\ NO_3^-$$

The reaction in the electrochemical side is described by

$$NO_3^-\,(aq) + 2H_2O\,(l) \rightarrow NH_3\,(g) + 2O_2\,(g) + OH^-\,(aq)$$

$$\Delta H^0 = 503.56\ kJ/mol\ of\ NH_3$$

$$\Delta G^0 = 411.956\ kJ/mol\ of\ NH_3$$

Therefore, the overall reaction for the pNOR-eNO_x^-RR system is

$$\frac{1}{2}N_2\,(g) + \frac{3}{2}H_2O\,(l) \rightarrow NH_3\,(g) + \frac{3}{4}O_2\,(g)$$

$$\Delta H^0 = 382.845\ kJ/mol\ of\ NH_3$$

$$\Delta G^0 = 339.267\ kJ/mol\ of\ NH_3$$

Supplementary Table 4. *Enthalpy (ΔH_f^o) and Gibbs free energy (ΔG_f^o) of the reactants and products.*

Compounds	ΔH_f^o (kJ/mol)	ΔG_f^o (kJ/mol)
N_2 (g)	0	0
O_2 (g)	0	0
H_2O (l)	−285.83	−237.178
OH^- (aq)	−229.6	−157.2
NO_3^- (aq)	−207.4	−111.3
NH_3 (g)	−45.9	−16.5

Hence, the energy efficiency for the pNOR-eNO_x^-RR tandem system is:

$$\eta = \frac{382.845}{18362.2} \times 100\% = 2.08\%$$

Supplementary Note 2. Detials for the DFT calculations.

All Gibbs free energy values were referenced to the computational hydrogen electrode (CHE) model using the proton-coupled electron transfer (PCET) approach[24,25]. The calculations were performed under conditions of pH = 0 and U (applied potential) = 0 V vs. the standard hydrogen electrode. The chemical potential of the H^+/e^- pair was considered as half of the H_2 gas molecule. The ΔG value was obtained as

$$\Delta \boldsymbol{G} = \Delta \boldsymbol{E}_{\boldsymbol{DFT}} + \Delta(\boldsymbol{ZPE} - \boldsymbol{TS})$$

where ΔE is the reaction energy difference between the product and the reactant occurring on catalysts. ΔZPE and ΔS are the changes in zero point energies and entropy at 298.15 K, which were calculated via considering only the vibrational frequencies. The Gibbs free energy of H* was calculated by

$$\Delta \boldsymbol{G}_{\boldsymbol{H}^*} = \Delta \boldsymbol{E}_{\boldsymbol{H}^*} + \Delta \boldsymbol{ZPE} - \boldsymbol{T}\Delta \boldsymbol{S}$$

where ΔS was obtained by

$$\Delta \boldsymbol{S} = \boldsymbol{S}(\boldsymbol{H}^*) - \frac{1}{2}\boldsymbol{S}(\boldsymbol{H}_2) \approx -\frac{1}{2}\boldsymbol{S}(\boldsymbol{H}_2)$$

This equation is obtained due to the negligible vibrational entropy of H*. At 300 K and 1 atm, $TS(H_2)$ = 0.41 eV; thus, TΔS = −0.205 eV[26].

The details for calculation of NO_3^- adsorption are described as follows.

The desired reaction for NO_3^- adsorption is

$$\boldsymbol{NO_3}^-(\boldsymbol{l}) + * \rightarrow \boldsymbol{NO_3}^* + \boldsymbol{e}^-$$

The thermodynamic cycle used to arrive at the required energetics is shown in Supplementary Figure 60. The overall free energy of nitrate adsorption is calculated as

$$\Delta \boldsymbol{G}_{\boldsymbol{ads}}(\boldsymbol{NO_3}^-) = \boldsymbol{G}_{\boldsymbol{NO_3}^*} + [\boldsymbol{G}_{\boldsymbol{H}^+} + \boldsymbol{G}_{\boldsymbol{e}^-}] - [\boldsymbol{G}_{\boldsymbol{NO_3}^-(\boldsymbol{l})} + \boldsymbol{G}_{\boldsymbol{H}^+}] - \boldsymbol{G}_*$$

where G_{NO3*} is the energy of NO_3^* adsorbed on the catalytic surface, $G_{NO3\text{-}(l)}$ is the free energy of aqueous NO_3^-. G_* is the energy of the respective surface, G_{e-} is the free energy of elementary charge and G_{H+} is the free energy of the proton. Here, we have neglected the rotational, translational, and vibrational free energies associated with adsorbed species, i.e., $G_{NO3*} = E_{NO3*}$, the value calculated from DFT. The zero-point energy correction for all species were not included.

Thus, the equation above can be converted into

$$\Delta \boldsymbol{G}_{\boldsymbol{ads}}(\boldsymbol{NO_3}^-) = \boldsymbol{G}_{\boldsymbol{NO_3}^*} + \frac{1}{2}\boldsymbol{G}_{\boldsymbol{H}_2} - [\boldsymbol{G}_{\boldsymbol{HNO_3}(\boldsymbol{g})} - \Delta \boldsymbol{G}_{\boldsymbol{vap}} - \Delta \boldsymbol{G}_{\boldsymbol{protonation}}] - \boldsymbol{G}_*$$

where G_{H2} and $G_{HNO3(g)}$ are the corresponding Gibbs free energies of H_2 and HNO_3 molecules in the gas phase at 300 K and 1 atm. The entropic (ΔS) and enthalpic (ΔH) contributions to the free energy of the gaseous species were obtained from JANAF thermodynamic tables[27]. ΔG_{vap} is the free energy of vaporization of HNO_3(l) and is calculated from the Gibbs free energy difference between standard formation of HNO_3 in liquid and gas phases and has a value of 0.075 eV. $\Delta G_{protonation}$ is the free energy of the association of NO_3^- in solution with a proton, and is calculated to be 0.317 eV[28]. These Gibbs free energies were all obtained from the CRC handbook of chemistry and physics[29]. Ultimately, the overall Gibbs free energy for NO_3^- adsorption from the solution phase ($\Delta G_{ads}(NO_3^-)$) was calculated as:

$$\Delta \boldsymbol{G}_{\boldsymbol{ads}}(\boldsymbol{NO_3}^-) = \boldsymbol{G}_{\boldsymbol{NO_3}^*} + \frac{1}{2}\boldsymbol{G}_{\boldsymbol{H}_2} - \boldsymbol{G}_{\boldsymbol{HNO_3}(\boldsymbol{g})} - \boldsymbol{G}_* + \mathbf{0.392\ eV} = \boldsymbol{E}_{\boldsymbol{NO_3}^*} + \frac{1}{2}\boldsymbol{E}_{\boldsymbol{H}_2} - \boldsymbol{E}_{\boldsymbol{HNO_3}(\boldsymbol{g})} - \boldsymbol{E}_* + \mathbf{0.75\ eV}$$

References

1 Ren, Y. *et al.* Microscopic-level insights into the mechanism of enhanced NH_3 synthesis in plasma-enabled cascade N_2 oxidation-electroreduction system. *J. Am. Chem. Soc.* **144**, 10193-10200 (2022).

2 Meng, S.-L. *et al.* Cobaloxime: selective nitrite reduction catalysts for tandem ammonia synthesis. *Energy Environ. Sci.* **16**, 1590-1596 (2023).

3 Sun, J. *et al.* A hybrid plasma electrocatalytic process for sustainable ammonia production. *Energy Environ. Sci.* **14**, 865-872 (2021).

4 Li, L. *et al.* Efficient nitrogen fixation to ammonia through integration of plasma oxidation with electrocatalytic reduction. *Angew. Chem. Int. Ed.* **60**, 14131-14137 (2021).

5 Li, W. *et al.* Sustainable nitrogen fixation to produce ammonia by electroreduction of plasma-generated nitrite. *ACS Sustain. Chem. Eng.***11**, 1168-1177 (2023).

6 Wu, A. *et al.* Direct ammonia synthesis from the air via gliding arc plasma integrated with single atom electrocatalysis. *Appl. Catal. B Environ.* **299** (2021).

7 Spry, M. *et al.* Water increases the faradaic selectivity of Li-mediated nitrogen reduction. *ACS Energy Lett.* **8**, 1230-1235 (2023).

8 Li, K. *et al.* Increasing current density of Li-mediated ammonia synthesis with high surface area copper electrodes. *ACS Energy Lett.* **7**, 36-41 (2021).

9 Cai, X. *et al.* Lithium-mediated electrochemical nitrogen reduction: Mechanistic insights to enhance performance. *iScience* **24**, 103105 (2021).

10 Lazouski, N., Chung, M., Williams, K., Gala, M. L. & Manthiram, K. Non-aqueous gas diffusion electrodes for rapid ammonia synthesis from nitrogen and water-splitting-derived hydrogen. *Nat. Catal.* **3**, 463-469 (2020).

11 Lazouski, N., Schiffer, Z. J., Williams, K. & Manthiram, K. Understanding continuous lithium-mediated electrochemical nitrogen reduction. *Joule* **3**, 1127-1139 (2019).

12 Suryanto, B. H. R., Matuszek, K., Choi, J. & Hodgetts, R. Y. Nitrogen reduction to ammonia at high efficiency and rates based on a phosphonium proton shuttle. *Science* **372**, 1187-1191 (2021).

13 Li, K. *et al.* Enhancement of lithium-mediated ammonia synthesis by addition of oxygen. *Science* **374**, 1593-1597 (2021).

14 Du, H. L. *et al.* Electroreduction of nitrogen with almost 100% current-to-ammonia efficiency. *Nature* **609**, 722-727 (2022).

15 Qiu, W. *et al.* High-performance artificial nitrogen fixation at ambient conditions using a metal-free electrocatalyst. *Nat. Commun.* **9**, 3485 (2018).

16 Yang, X. *et al.* Mechanistic insights into electrochemical nitrogen reduction reaction on vanadium nitride nanoparticles. *J. Am. Chem. Soc.* **140**, 13387-13391 (2018).

17 Lin, Y. X. *et al.* Boosting selective nitrogen reduction to ammonia on electron-deficient copper nanoparticles. *Nat. Commun.* **10**, 4380 (2019).

18 Liu, S. *et al.* Facilitating nitrogen accessibility to boron-rich covalent organic frameworks via electrochemical excitation for efficient nitrogen fixation. *Nat. Commun.* **10**, 3898 (2019).

19 Luo, Y. *et al.* Efficient electrocatalytic N_2 fixation with MXene under ambient conditions. *Joule* **3**, 279-289 (2019).

20 Chu, K. *et al.* Unveiling the synergy of O^- vacancy and heterostructure over MoO_{3-x}/MXene for N_2 electroreduction to NH_3. *Adv. Energy Mater.* **12** (2021).

21 Liu, S. *et al.* Proton-filtering covalent organic frameworks with superior nitrogen penetration flux promote ambient ammonia synthesis. *Nat. Catal.* **4**, 322-331 (2021).

22 Zhou, G. *et al.* Recharged catalyst with memristive nitrogen reduction activity through learning networks of spiking neurons. *J. Am. Chem. Soc.* **143**, 5378-5385 (2021).

23 Kang, J. *et al.* Activating Bi p-orbitals in dispersed clusters of amorphous BiO_x for electrocatalytic nitrogen reduction. *Angew. Chem. Int. Ed.* e202217428 (2023).

24 Karlberg, G. S. *et al.* Cyclic voltammograms for H on Pt (111) and Pt (100) from first principles. *Phys. Rev. Lett.* **99**, 126101 (2007).

25 Nørskov, J. K. et al. Origin of the Overpotential for Oxygen Reduction at a Fuel-Cell Cathode. *J. Phys. Chem. B* **108,** 46, 17886–17892 (2004).

26 Li, Q. et al. Revealing activity trends of metal diborides toward pH-universal hydrogen evolution electrocatalysts with Pt-like activity. *Adv.Energy Mater.* **9,** 1803369 (2019).

27 Allison, T. C. NIST-JANAF Thermochemical Tables - SRD 13. *National Institute of Standards and Technology,* (2013).

28 Liu, J. X., Richards D., Singh N. & Goldsmith B. R. Activity and Selectivity Trends in Electrocatalytic Nitrate Reduction on Transition Metals. *ACS Catal.* **9,** 7052–7064 (2019).

29 Lide, D. R. CRC handbook of chemistry and physics. *CRC press,* (2004).
